# Multi-Omics Analysis of Mammary Metabolic Changes in Dairy Cows Exposed to Hypoxia

**DOI:** 10.3389/fvets.2021.764135

**Published:** 2021-10-14

**Authors:** Zhiwei Kong, Bin Li, Chuanshe Zhou, Qinghua He, Yuzhong Zheng, Zhiliang Tan

**Affiliations:** ^1^Department of Food Science and Engineering, College of Chemistry and Environmental Engineering, Shenzhen University, Shenzhen, China; ^2^Key Laboratory of Optoelectronic Devices and Systems of Ministry of Education and Guangdong Province, College of Optoelectronic Engineering, Shenzhen University, Shenzhen, China; ^3^School of Food Engineering and Biotechnology, Hanshan Nornal University, Chaozhou, China; ^4^State Key Laboratory of Hulless Barley and Yak Germplasm Resources and Genetic Improvement, Institute of Animal Husbandry and Veterinary, Tibet Autonomous Regional Academy of Agricultural Sciences, Lhasa, China; ^5^CAS Key Laboratory for Agro-Ecological Processes in Subtropical Region, National Engineering Laboratory for Pollution Control and Waste Utilization in Livestock and Poultry Production, Hunan Provincial Key Laboratory of Animal Nutritional Physiology and Metabolic Process, Institute of Subtropical Agriculture, Chinese Academy of Sciences, Changsha, China

**Keywords:** metabonomics, lipidomics, hypoxia, lipid metabolism, dairy cow

## Abstract

Hypoxia exposure can cause a series of physiological and biochemical reactions in the organism and cells. Our previous studies found the milk fat rate increased significantly in hypoxic dairy cows, however, its specific metabolic mechanism is unclear. In this experiment, we explored and verified the mechanism of hypoxia adaptation based on the apparent and omics results of animal experiments and *in vitro* cell model. The results revealed that hypoxia exposure was associated with the elevation of AGPAT2-mediated glycerophospholipid metabolism. These intracellular metabolic disorders consequently led to the lipid disorders associated with apoptosis. Our findings update the existing understanding of increased adaptability of dairy cows exposure to hypoxia at the metabolic level.

## Introduction

Mammary gland has the special function of secreting milk and is mainly made up of mammary epithelial cells (MECs). A lot of components in milk can only be synthesized by MECs. The number and activity of mammary epithelial cells reflect the lactation ability of mammary gland (1, 2). Therefore, mammary epithelial cells play an important role in mammary development and lactation. In high-yield dairy cows, a large number of milk production and secretion will cause spikes in energy demand of mammary cells. Thus, its aerobic metabolism activity is significantly enhanced by regulating lipid metabolism (3), leading to hypoxia (determined by arteriomammary venous O_2_ and CO_2_ level) in the mammary internal environment (4).

Hypoxia is a pathological process involved in a variety of physiological and biochemical changes (5). Hypoxia can cause cell damage, including cell apoptosis, oxidative stress, mitochondrial dysfunction and abnormal lipid metabolism (6). Marques et al. (7) found that increasing lipid storage and inhibiting lipid catabolism could adapt to hypoxia stress. Under hypoxia, lipid storage is mediated by hypoxia inducible factor 1α (HIF1α), and lipid levels are increased by regulating the expression of peroxidase in lipid metabolism (8). The inhibition of hypoxic lipolysis is mainly achieved by reducing the expression of PPAR γ 2 (7) and inhibiting fatty acid β-oxidation (9). As one of the main substances in milk, milk fat is mainly composed of triglycerides (more than 95%) synthesized from fatty acids and a small amount of other lipids (10, 11). At present, the research on the regulation mechanism of milk fat synthesis mostly focuses on the effects of genetics (12), environment (13), hormone levels (14), and nutritional status (15). However, the mechanism of milk fat synthesis of dairy cow in hypoxic condition has not been elucidated.

Metabolomics based on chromatography and mass spectrometry is a research method to search for the relative relationship between small molecule metabolites and physiological and pathological changes (16). At present, studies on metabolic changes under environmental stress have been carried out in many animals (17, 18). Lipidomics is becoming an important research field based on the development of mass spectrometry and bioinformatics (19). Although some researchers have used these methods to explore the lipid response of dairy cows under biological stress (20), and metabonomics and lipidomics can be used as powerful tools to identify new signaling pathways in hypoxic stress (21, 22), our understanding of lipid responses to hypoxic stressor is still limited. In our previous studies, the results showed that the level of milk fat increased under hypoxia. Therefore, we speculated that the increase of milk fat level was caused by hypoxia through regulating lipid metabolism. In order to test this hypothesis, we first detected the serum biochemical indicators and milk quality, and then constructed a hypoxia stress model of bovine mammary epithelial cells (BMECs) *in vitro*, and finally carried out metabonomics and lipidomics analysis. We found that acylglycerol-3-phosphate acyltransferase 2 (AGPAT2)-mediated glycerophospholipid metabolism played an important role in the process of hypoxia induced increase in lipid synthesis. This will help us to understand the mechanism of hypoxia adaptation more comprehensively.

## Materials and Methods

The present study was carried out based on the Animal Care and Use Guidelines of the Animal Care Committee, Institute of Subtropical Agriculture, Chinese Academy of Sciences, Changsha, China, with protocol ISA-201710.

### Animals and Experimental Design

Twelve multiparous Holstein dairy cows (600 ± 35 kg) were chosen and randomly divided into two groups in Shenyang (GH) (average altitude 50 m). One group (six cattle in each group) was selected and raised in Nyingchi (CH) in autumn (average altitude 3,000 m) for 30 days. Another group was raised in Shenyang at the same time for 30 days. The experimental animals were in good physiological condition, and they were not pregnant cows, but were in early lactation (the third month after delivery). The animals were raised in a single column to reduce the stress caused by excessive exercise.

The TMR basal diet (Table 1) is prepared according to the Feeding Standards of Dairy Cattle in China, which can meet the energy, protein, minerals and vitamins required for basic metabolism of dairy cows (23). All dairy cows were freely supplied the same TMR diet.

**Table 1:** Ingredient composition and nutrition levels of the diet (% of DM).

**Items**	**Content (%)**
**Diet composition**	
Chinese leymus	37.5
Corn silage	22.5
Corn	15.2
Wheat bran	5.3
Soybean meal	9.2
DDGS	8.4
**Calcium hydrophosphate**	1.4
Premix^a^	0.5
Nutrient composition	
CP	13.1
NDF	39.6
Ca	0.6
P	0.4
NEL^b^, MJ/kg DM	5.4

a *One kilogram of premix contained mixed vitamins, 800,000 IU; Fe, 1,500 mg; Cu, 1,000 mg; Zn, 11,000 mg; Mn, 3,500 mg; Se, 80 mg; I, 200 mg; and Co, 50 mg*.

b *NEL was calculated. DDGS, distillers dried grains with soluble; CP, crude protein; DNF, neutral detergent fiber; NEL, Net energy of Lactation*.

### Sample Preparation

On the last day morning, all experimental animals were punctured through the caudal vein and collected blood into K2 EDTA anticoagulant vacuum tube before the feeding. Plasma was separated from blood samples by centrifugation at 3,000 g for 10 min, and then collected and stored at −80°C for subsequent metabolomic analysis. Serum samples were collected from blood at 4,000 rpm for 5 min and stored at −40°C for subsequent detection of biochemical indicators. Milk samples (10 mL) were collected on the morning and evening of the last day of this experiment. The collected milk samples were stored in a −20°C freezer for milk fat level detection.

### Analysis of the Serum Biochemical Indicators and Milk Quality

The levels of triglyceride, total cholesterol and high-density lipoprotein were measured by Enzyme-Linked Immunosorbent Assay (ELISA) Kits (Sekisui Diagnostic Ltd., Stamford, CT, USA) through an automatic blood analyzer (Hitachi 7170A, Japan). Milk fat was detected by Basic Unit MilkoScan FT 76150 (FOSS, Denmark).

### Cell Culture and Treatment

BMECs were isolated and cultured based on the methods described previously (24). In brief, Bard Magnum biopsy gun and biopsy needle were selected as the tools for udder tissue collection. Fifty to one hundred mg of tissue from 2-year-old late-lactation dairy cows were collected from the midpoint of the upper quarter of the posterior area of the udder 3 h after milking by No. 12 biopsy needle. The samples was harvested and transferred to the lab immediately. The samples were washed 3 times with DPBS (D8662, sigma, USA), and then transferred to clean cell culture dishes with tweezers. The tissue was cut into 1mm^3^ pieces with surgical tweezers. The tissue blocks were evenly daubed on the bottom of the cell culture dish and placed upside down in the carbon dioxide cell incubator for 2–4 h. Then appropriate complete medium [including DMEM/F12 (12400-024; Gibco, USA), 10% fetal bovine serum (FBS; Sigma-Aldrich, St. Louis, MO), penicillin/streptomycin (Baoman Biotechnology, Shanghai, China), and 4 μg/ml prolactin (Sigma-Aldrich)] was added for culture. When the epithelial cells reached about 80% of the bottom of the culture dish, the cells were subcultured with 0.25% trypsin. The cells were purified by differential adhesion and then cryopreserved (25). Purified cells were counted and inoculated in culture dishes. When the cells reached 70–80% confluence, they were treated with hypoxia. The medium was changed every 2–3 d. BMECs were exposed to hypoxia (1% O_2_) for 72 h. Cells cultured in an incubator with normoxia (21% O_2_) were served as controls.

### siRNA Transfection

AGPAT2 siRNA library for various parts of AGPAT2 mRNA and negative control siRNA (NC) were designed and provided by Ribo Bio (Guangzhou, China). The siRNA sequence of AGPAT2 applied was shown as following: siRNA-1: 5′-CTACCGTTGTTATAGGTG-3′ (control), siRNA-2: 5′-GGAGAATCTCAAAGTGTGG-3′, siRNA-3: 5′-TGTCAAGACGAAGCTCTTC-3′. BMECs were transfected with 2 mg of the AGPAT2 siRNA or NC siRNA in 10 mL X-tremeGENE siRNA Transfection Reagent (03366236001, Roche). Twenty-four hours after transfection, the cells were collected and used for subsequent index detection.

### Determination of Triglyceride (TAG) Content in Cells

BMECs were pretreated with hypoxia for 72 h. TAG levels were determined as described previously (26). Briefly, the treated cells were firstly digested by trypsin and collected, and then the triglyceride test solution was extracted according to the instructions of the triglyceride detection kit (Applygen, Beijing, China). Finally, the microplate reader (BIORAD, CA, USA) was adjusted to the appropriate wavelength and the triglyceride content was calculated according to the OD value.

### Oil Red O (ORO) Staining

BMECs were pretreated with hypoxia for 72 h. The formation of lipid droplets was observed according to the method previously described (27). Briefly, the treated BMECs cells were fixed with 10% neutral formaldehyde fixing solution (Sigma-Aldrich, St. Louis, MO, USA), stained with ORO (Sigma-Aldrich), then rinsed and decolorized with 75% alcohol/60% isopropanol, and finally re-stained with light hematoxylin (Sigma-Aldrich) and sealed with glycerol gelatin for observation and photography under the microscope (Olympus, Tokyo, Japan).

### Fluoroboron Dipyrrole (BODIPY) Staining

After hypoxia treatment, the cells were collected and fixed with 4% formaldehyde (Sigma-Aldrich), then rinsed using phosphate buffer, stained by BODIPY493/503 (Thermo Fisher Scientific, MA, USA), and then covered with tablet sealant containing DAPI. Finally, confocal laser microscope was used to take photos and observe the distribution of lipid droplets.

### Real-Time PCR

After treatment of hypoxia for 72 h, the level of genes related to milk fat synthesis were determined as described previously (28). In brief, 1 × 10^6^~1 × 10^7^ cells were taken from each sample, and the cells were washed with PBS. PBS was removed and 1 mL of RNA extraction reagent Trizol (Invitrogen) was added. RNA was extracted after lysis. Genomic DNA was cleared by DNaseI (Thermo Scientific), and the quality and quantity of RNA obtained were evaluated by Nano Drop 2000 (Thermo Scientific). Primers were designed using Primer 5.0 software, and PCR Primer sequences were shown in Table 2. The PCR amplification conditions were as follows: pre-denaturation at 95°C for 30 s; after denaturation at 95°C for 5 s, each gene was annealed at the optimum annealing temperature for 20 s and 72°C for 20 s, a total of 40 cycles were carried out. After PCR reaction, the melting curve was drawn to judge the correctness of the amplified products. The temperature raised from 60 to 95°C at the rate of 0.5°C/5 s. Using β-actin as internal reference, the CT values of each sample were homogenized. Under the condition that the amplification efficiency of each target gene and β-actin was basically the same, the expression levels of related genes were compared and analyzed by 2^−ΔΔCT^ using the gene expression level of control group as the reference (29).

**Table 2:** Gene primers.

**Gene Name**	**GeneBank No**.	**Primer Sequence (5^**′**^-3^**′**^)**	**Product Size (bp)**
		GCAGCCCCTCAAGCGAACAGT	
*FASN*	NM_001012669.1		123
		ACCGCCTCCTGCTCTTCCTCACGTAA	
		CTCTTTGTTTGGTCGTGATTGCTCT	
*ACACA*	NM_174224.2		126
		CTGGCAAGTTTCACCGCACAC	
		CCAACAACTCTGCCTTTATGATGC	
*SCD1*	NM_173959.4		155
		TGACTGACCACCTGCTTGCC	
		CCCTGCAAAACACAGACCCA	
*CD36*	NM_001278621.1		178
		ATGGTTATAATGCCTTGCTGATGCT	
		ACCAAGCCTACCACAATCATCG	
*FABP3*	NM_174313.2		170
		ACAAGTTTGCCGCCATCCAG	
		AGCGAGAACATCCCTTTTACCCT	
*LPL*	NM_001075120.1		91
		GCAATTCTCCAATATCCACCTCCGT	
		TTCCCAAGAGCTGACCCGAT	
*PPARG*	NM_181024.2		185
		TCCCCACAGACCCGGCAT	
		ACAGCCCACAACGCCATCGAG	
*SREBP1*	NM_001113302.1		248
		CCTCCACTGCCACAAGCCGACAC	
		ACTGTTAGCTGCGTTACACC	
*β-actin*	NM_173979.3		167
		TGCTGTCACCTTCACCGTTC	

*FASN, fatty acid synthase; ACACA, acetyl-CoA carboxylases alpha; SCD1, stearoyl coenzyme A1; CD36, Fatty acid synthase subunit beta; FABP3, fatty acid binding protein 3; LPL, lipoproteinlipase; SREBP1, sterol regulatory element binding protein1; PPARG, peroxisome proliferator-activated receptor gamma*.

### Western Blotting Analysis

After treatment of hypoxia for 72 h, the protein isolation and western blotting of BMECs were conducted based on previous methods (30, 31). Briefly, in 10% SDS-polyacrylamide gels, electrophoresis separation was performed for each sample and pre-stained standard (Bio-RAD Laboratories, Berkeley, CA, USA). The polyvinylidene fluoride (PVDF) membranes (Bio-RAD Laboratory) containing isolates were firstly incubated with primary antibodies (see Table 3) at 4°C overnight, washed and then incubated in a blocking solution with secondary antibody (1:6,000, Proteintech) at 25°C for 2 h. The images were taken and analyzed by a Alpha Imager 2200 digital imaging system (Digital Imaging System, Kirchheim, Germany).

**Table 3:** Details of the primary antibodies.

**Name**	**No**.	**Host species**	**Dilution**	**Supplier**
*PPARG*	ab45036	Rabbit	1:500	Abcam
*SREBP1*	14088-1-AP	Rabbit	1:1,000	Proteintech
β-actin	66009-1-Ig	Mouse	1:5,000	Proteintech

*PPARG, peroxisome proliferator-activated receptor gamma; SREBP1, sterol regulatory element binding protein1*.

### Metabolomics and Lipidomics Analysis

One hundred microliters of blood samples/BMECs were collected. Three hundred microliters methanol (including internal standard) was added to the sample, and protein precipitation was obtained by vortices. Samples after vortex were then centrifuged at 12,000 rpm for 15 min. Finally, transfer the supernatant to LC-MS loading bottle for storage at −80°C for UHPLC-QE Orbitrap/MS analysis (metabolomics). Cells samples were homogenized with MTBE and sonicated in ice-water bath for 5 min. Then the sonicated samples were centrifuged at a rate of 3,000 rpm at 4°C for 15 min. Three hundred microliters of supernatant was taken and dried. Then, the dried samples were reconstituted and centrifuged. Appropriate amount of the recombinant supernatant was put into a new sample bottle for LC/MS analysis (lipidomics).

LC-MS/MS Analysis for untargeted metabolomics: UHPLC system (1290, Agilent Technologies) combined Q exactive (Orbitrap MS, thermo) performed the LC-MS/MS analysis for this trial. The mobile phase A used in the instrument is 0.1% formic acid aqueous solution and acts as a positive. Mobile phase B is acetonitrile, while ammonium acetate aqueous solution acts as negative. The sample size required for the test is 3 μl. The characteristic of the mass spectrum is to obtain the MS/MS spectrum in an information-dependent basis (IDA).

LC-MS/MS Analysis for untargeted lipidomics: the UPLC system used in this test is unique in that it is equipped with kinetex C18 column and Exionlc infinity system. Mobile phase A (positive) is a mixture of water, acetonitrile and ammonium formate, while mobile phase B (negative) is a mixture of acetonitrile, isopropyl alcohol and ammonium formate. The loading quantity is 2 μl. Spectra were obtained using a TripleTOF 5600 mass spectrometer.

For data processing of metabonomics, the original data is converted into mzXML format by proteowizard and processed by internal program. The program is developed by R and based on xcms for peak detection, extraction, comparison, and integration. The MS2 internal database (BiotreeDB) was then used for metabolite annotation. The cutoff value for comments is set to 0.3. For data processing of lipidomics, the raw data files (.wifff format) have been converted to mzXML format through the msConvert program in the Proteowizard. Firstly, the CentWave algorithm in XCMS was used to detect the peak value of MS1 data, and then the obtained MS/MS spectra were matched with LipidBlast library to obtain the lipids screened in the experiment.

### Statistical Analysis

SPSS software is mainly applied for data analysis. The levels of triglyceride in cells, relative mRNA expression of genes associated with milk fat synthesis and protein abundance were analyzed using one-way ANOVA. The data are shown as the mean ± SD.

Q Exactive Orbitrap (Thermo Fisher Scientific, USA) and Ultra High Performance Liquid Tandem Chromatography Quadrupole Time of Flight Mass Spectrometry (UHPLC-QTOFMS, AB Sciex, USA) were applied to analyze the data of metabolomics and lipidomics. Principal component analysis is conducted on the normalized original data to observe the reliability of the data. Orthogonal partial least squares discriminant analysis (OPLS-DA) was used to filter out the non-conforming metabolites. Univariate statistical analysis (UVA) was used to screen the differential metabolites [*P* < 0.05, and Variable Importance in the Projection (VIP) > 1] and make the volcanic map. KEGG, PubChem and other authoritative metabolite databases were used to analyze the metabolic pathways of differential metabolites. Self-built database from Shanghai Biotree biotech CO., Ltd. was used to lipidomics analysis.

## Results

### Serum Lipid Metabolism Related Indexes and Milk Fat Level

**Figure 1:**
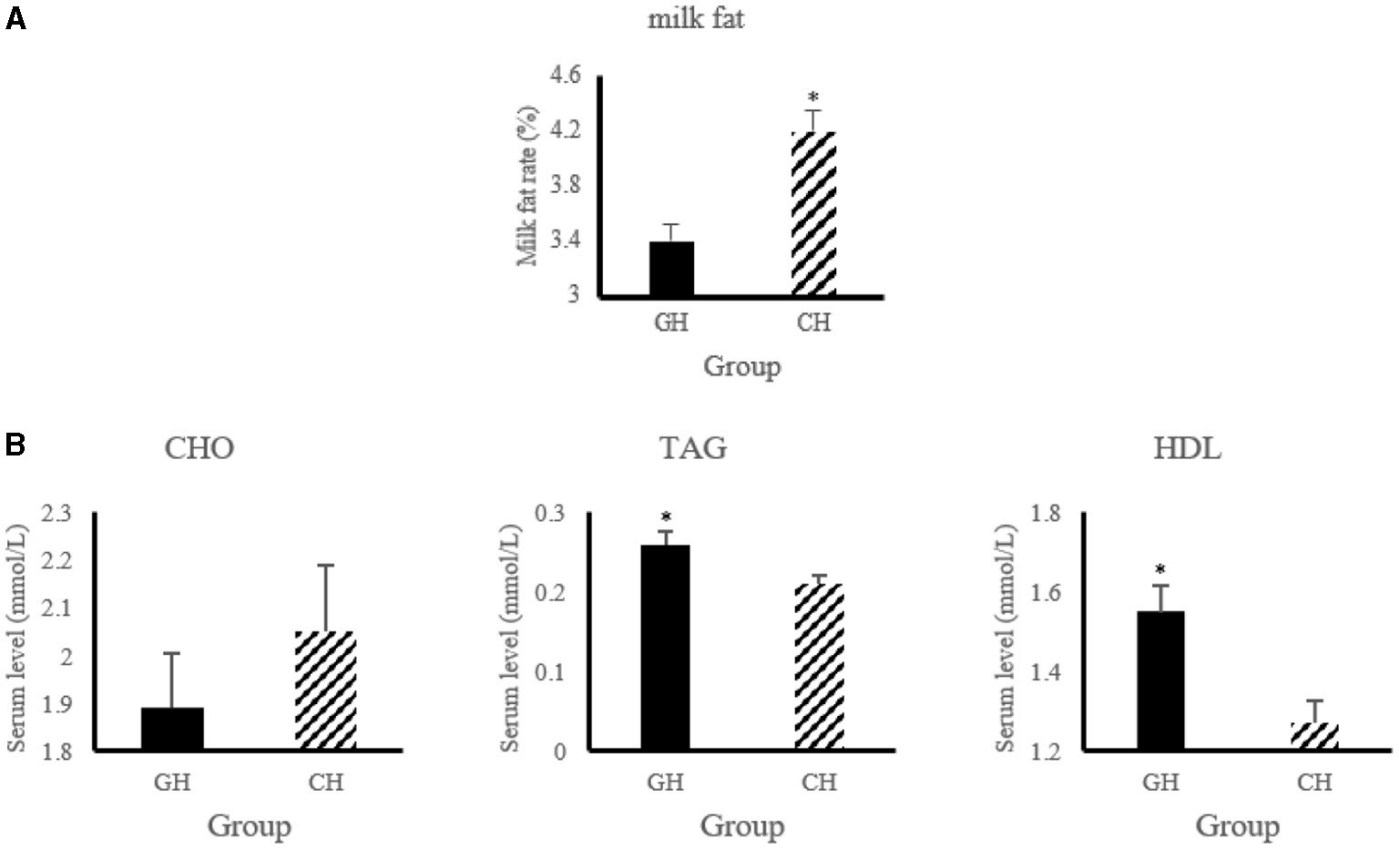
Lipid metabolic changes of altered by hypoxia exposure in milk and blood of dairy cows. **(A)** Changes of milk fat and protein. “GH and CH” represent control, hypoxia group, respectively; **(B)** Changes of serum biochemical indexes. CHO, Cholesterol; TAG, triglycerides; HDL, high-density lipoprotein. *n* = 6, *p < 0.05.

As shown in Figure 1, the level of serum triglycerides and high-density lipoprotein increased (*P* < 0.05) in hypoxia-stressed dairy cows compared with hypoxia-free dairy cows. In addition, the dairy cows in hypoxia group had higher (*P* < 0.05) milk fat level than that of the control group.

### Plasma Metabolomics Analysis of Hypoxic Dairy Cows

**Figure 2:**
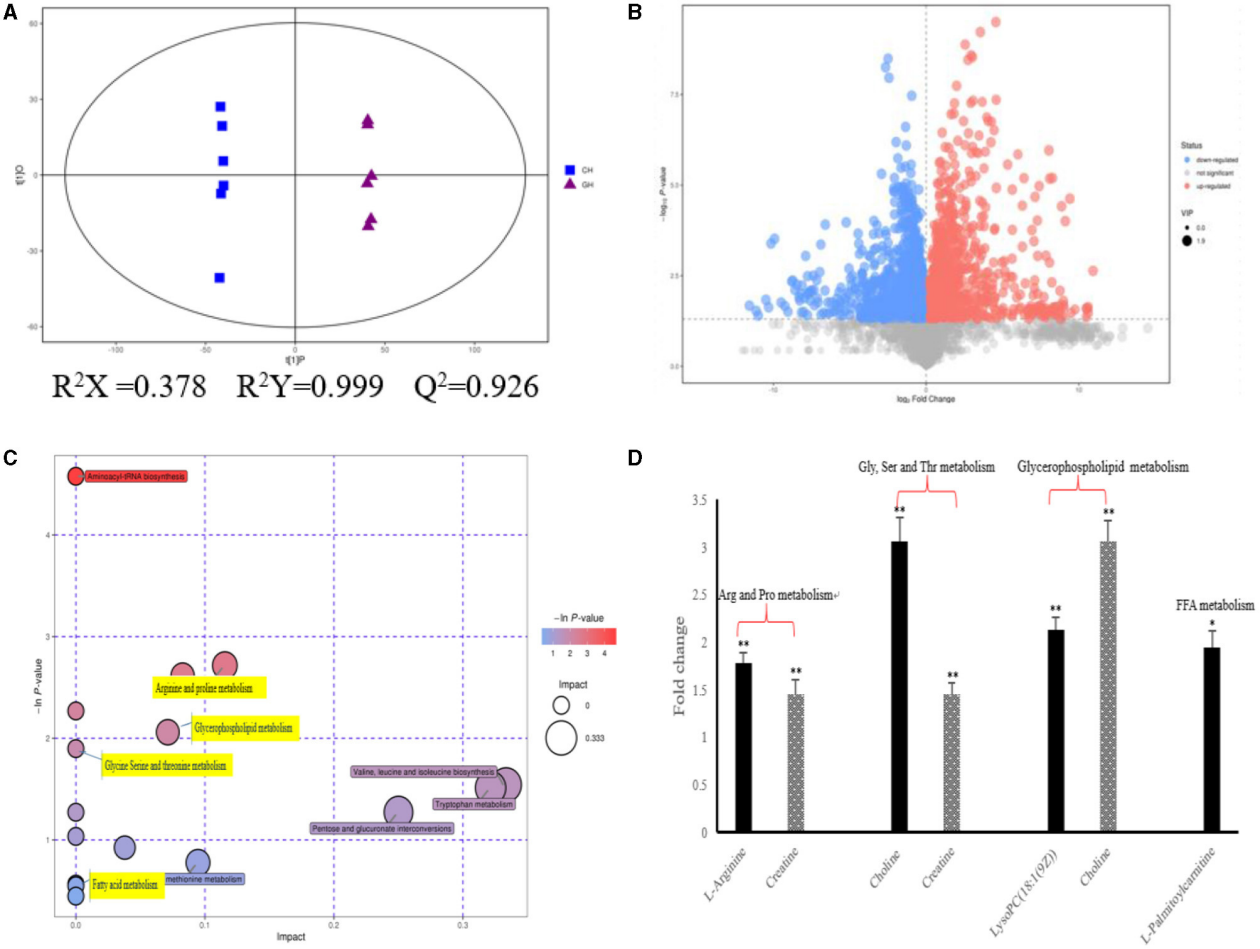
Metabolic changes altered by hypoxia exposure in plasma of dairy cows. **(A)** PLS-DA score plot of the two groups in plasma of dairy cows using the identified metabolites in positive ionization mode. “GH and CH” represent control, hypoxia group, respectively; **(B)** Volcanic plots of the two groups in plasma of dairy cows using the identified metabolites in positive ionization mode; **(C)** Pathway analysis of the identified metabolites in plasma; **(D)** The fold changes of the significantly changed metabolites of plasma in metabolic pathways in hypoxia exposure group relative to the control group. Arg, Arginine; Pro, proline; Gly, Glycine; Ser, serine; Thr, threonine; Val, Valine; Leu, leucine; Ile, isoleucine; FFA, Free Fatty acid. *n* = 6, *p < 0.05, **p < 0.01.

As shown in Figure 2, we could observe the metabolic changes induced by hypoxia exposure in plasma of dairy cows. In positive ionization mode, 4,083 metabolic characteristics of plasma were extracted to establish PLS-DA model (Figure 2A). Goodness of fit (R^2^Y) and predictive power (Q^2^) are often used to verify PLS-DA model. The R^2^Y and Q^2^ values of the PLS-DA model are both >0.9, which indicates that the PLS-DA model has good fitting and strong prediction ability. In combination with the results of the volcanic plots (Figure 2B), we found that hypoxia exposure interfered with plasma metabolism in dairy cows. Through MS2 spectral matching, 96 potential biomarkers were identified (Supplementary Table 1; Supplementary Material) in plasma, including amino acids, peptides, nucleosides, nucleotides, and phospholipids. In addition, metaboanalyst (4.0) based on KEGG database was used to analyze the most relevant metabolic pathways of hypoxia exposure changes (Figure 2C). From the results, the up-regulated pathways were arginine and proline metabolism, glycine, serine and threonine metabolism, and glycerophospholipid metabolism, while the down regulated pathways were fatty acid metabolism (Figure 2C), which were involved in metabolic disturbance.

### Hypoxia Contributes to Lipid Synthesis in BMECs

**Figure 3:**
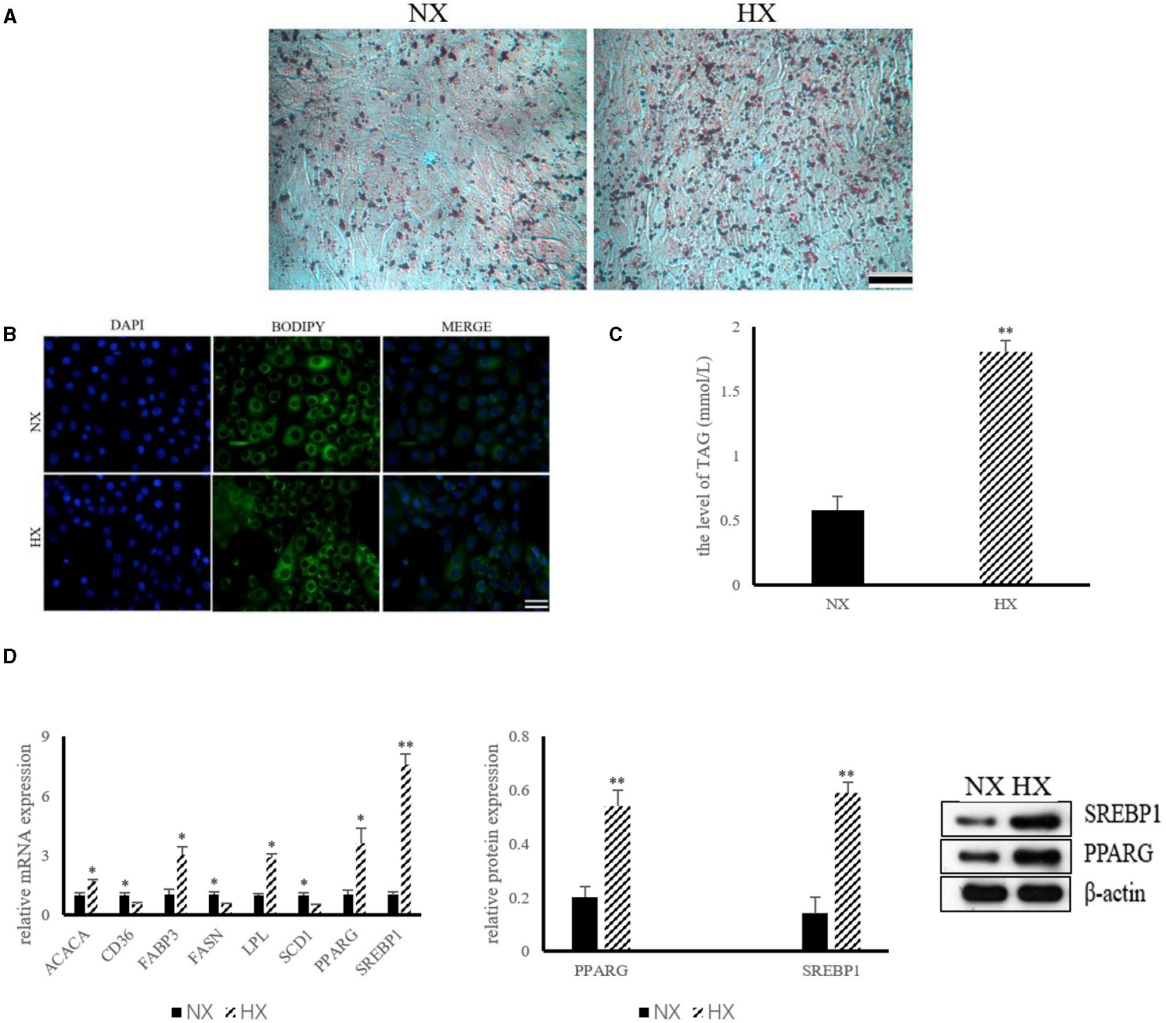
Lipid synthesis by hypoxia exposure in BMECs. **(A)** ORO staining of BMECs under hypoxia. NX, normoxia; HX, hyoxia; **(B)** BODIPY staining of BMECs under hypoxia; **(C)** Level of TAG in BMECs under hypoxia; **(D)** Expression of genes and proteins related to lipid synthesis under hypoxia. *n* = 3, *p < 0.05, **p < 0.01. FASN, fatty acid synthase; ACACA, acetyl-CoA carboxylases alpha; SCD1, stearoyl coenzyme A1; CD36, Fatty acid synthase subunit beta; FABP3, fatty acid binding protein 3; LPL, lipoproteinlipase; SREBP1, sterol regulatory element binding protein1; PPARG, peroxisome proliferator-activated receptor gamma.

The results of lipid synthesis induced by hypoxia are shown in Figure 3. The results of ORO and BODIPY staining indicated that hypoxia could promote the formation of lipid droplets in BMECs (Figures 3A,B). The level of TAG in hypoxia group was significantly higher (*P* < 0.05) than that of control group (Figure 3C). In addition, compared with the control group, the mRNA expressions of ACACA, FABP3, LPL, PPARG, and SREBP1 increased significantly (*P* < 0.05), while the levels of CD36, FASN and SCD1 decreased (*P* < 0.05) significantly (Figure 3D). The protein abundance of PPARG and SREBP1, the key protein of hypoxia cell lipid synthesis, was also increased (*P* < 0.05) significantly (Figure 3D).

### Cell Metabolomics Analysis

**Figure 4:**
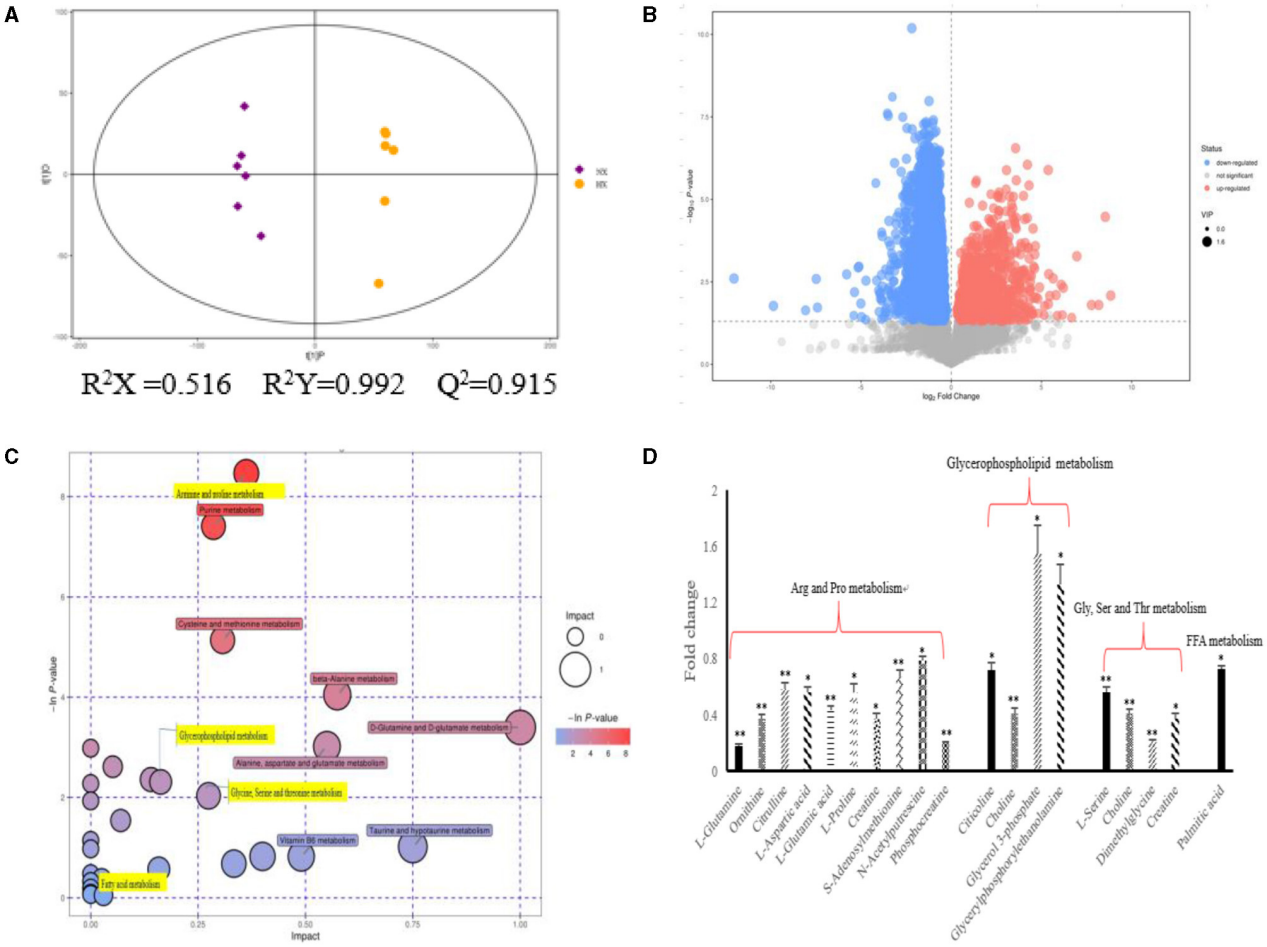
Metabolic changes altered by hypoxia exposure in BMECs of dairy cows. **(A)** Two groups of PLS-DA scores were performed on BMECs using the identified metabolites under positive ionization mode. “NX” and “HX” represent normoxia and hypoxia groups, respectively; **(B)** Volcanic plots of the two groups in BMECs using identified metabolites under positive ionization modes; **(C)** Pathway analysis of identified metabolites in BMECs; **(D)** Fold changes in metabolites significantly altered in the metabolic pathway of BMECs in the HX group compared to the NX group. Arg, Arginine; Pro, proline; Gly, Glycine; Ser, serine; Thr, threonine; Val, Valine; Leu, leucine; Ile, isoleucine; FFA, Fatty acid. *n* = 6, *p < 0.05, **p < 0.01.

As shown in Figure 4, we could observe the metabolic changes induced by hypoxia exposure in BMECs. In positive ionization mode, 3,361 metabolic characteristics of BMECs were extracted to establish PLS-DA model (Figure 4A). The R^2^Y and Q^2^ values of the PLS-DA models are both >0.9, which shows that hypoxia exposure can interfere with BMECs metabolism. The results in volcanic plots (Figure 4B) also showed the same condition as shown in Figure 4A. Through MS2 spectral matching, 302 potential biomarkers such as amino acids, peptides, nucleosides, nucleotides, and phospholipids were identified (Supplementary Table 2; Supplementary Material). In addition, the up-regulated pathways including arginine and proline metabolism, glycine, serine and threonine metabolism, and glycerophospholipid metabolism and down-regulated pathways such as fatty acid metabolism (Figure 4C) were screened out by metaboanalyst (4.0) based on KEGG database.

### Cell Lipidomics Analysis

**Figure 5:**
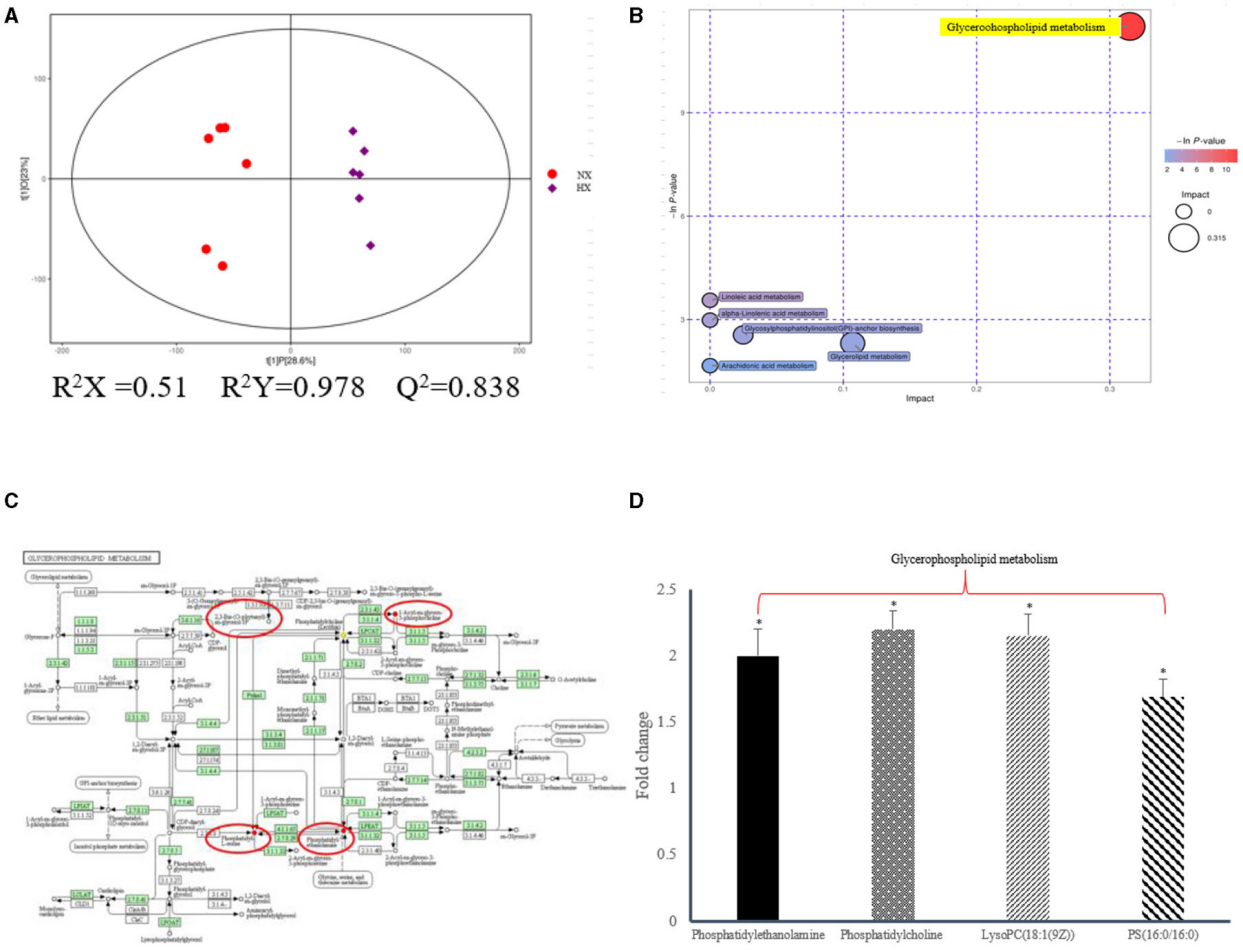
Lipidomic changes altered by hypoixa exposure in BMECs. **(A)** PLS-DA score plot of the two groups of BMECs using the differential metabolites under positive ionization mode. “NX and HX” represent normoxia and hypoxia group, respectively; **(B)** Pathway analysis of the differential metabolites; **(C)** KEGG enrichment analysis of diffential metabolites; **(D)** Fold changes of the significantly changed metabolites in glycerophospholipid metabolic pathways in HX group relative to NX group. *n* = 6, *p < 0.05.

In the current research, non-targeted HPLC-QTOF-MS was used to investigate the differential expression of lipid metabolites in hypoxic and normoxic BMECs with high sensitivity, specificity and peak resolution (Figure 5). After signal standardization, lipids identified from positive and negative ionization modes were introduced into SIMCA-P to establish the PLSDA model (Figure 5A; Supplementary Figure 1; Supplementary Material). The R^2^Y and Q^2^ values of the two PLS-DA models were both >0.8, which indicated that the model had good fitting and strong prediction ability. We have demonstrated that the differential regulation of glycerophospholipid metabolites (Figures 5B,C) had a strong correlation with the diagnosis and prognosis of hypoxia, and a total of 541 lipids (Supplementary Figure 2; Supplementary Material) were screened, in which 27 phosphatidylethanolamines (PEs), 9 phosphatidylserines (PSs), 31 phosphatidylcholine (PCs), and 3 phosphatidylglycerols (PGs) were up-regulated. Oppositely, the levels of 3 PCs decreased (Figure 5D). The details of the differential lipids were shown in Supplementary Table 3; Supplementary Material.

### Cell Apoptosis Detection

**Figure 6:**
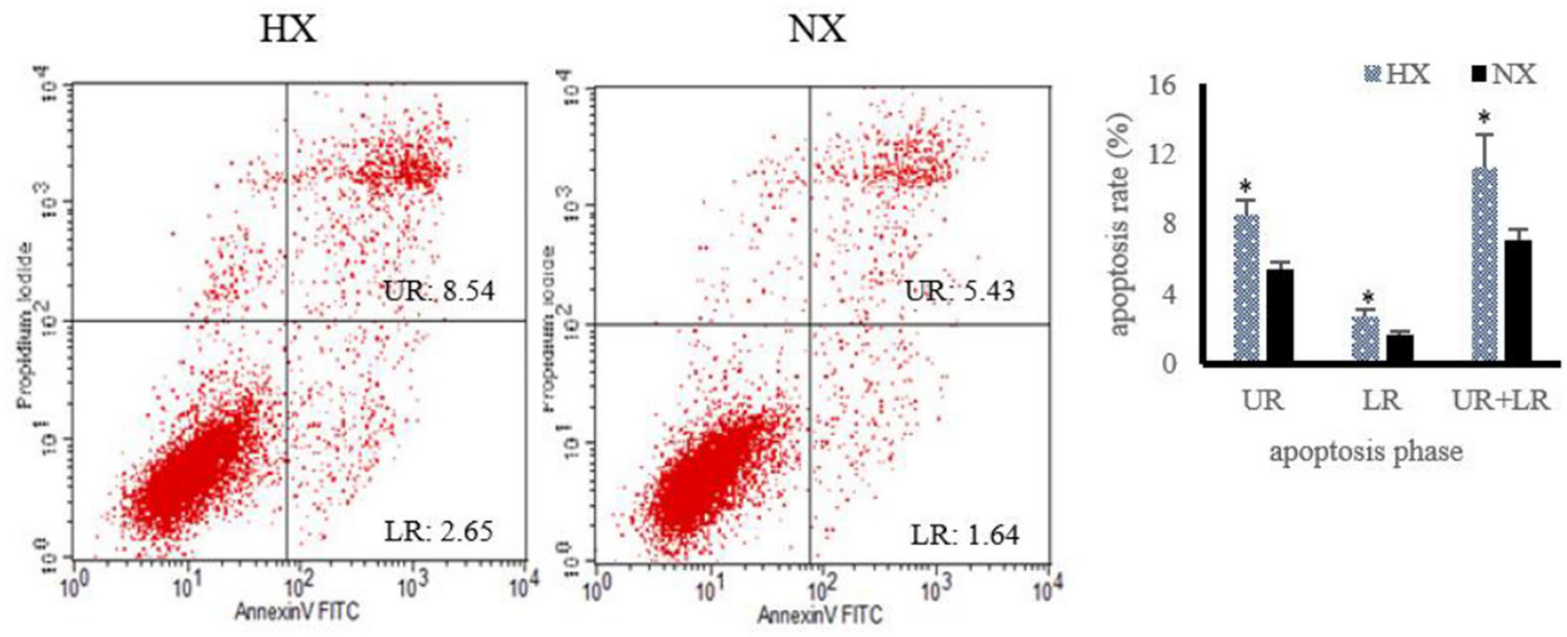
Changes of cell apoptosis after hypoxia exposure (*n* = 3). Apoptosis rate under hypoxia detected by flow cytometry, “HX and NX” represent hypoxia, normoxia group; The data are expressed as mean ± SD. Compared with the control, *p < 0.05 show that the difference is statistically significant.

The changes of cell apoptosis after hypoxia exposure are shown in Figure 6. Compared with normoxic BMECs, the apoptosis rate of hypoxic BMECs increased (*P* < 0.05) significantly.

### Verification the Role of Glycerophospholipids Metabolism

**Figure 7:**
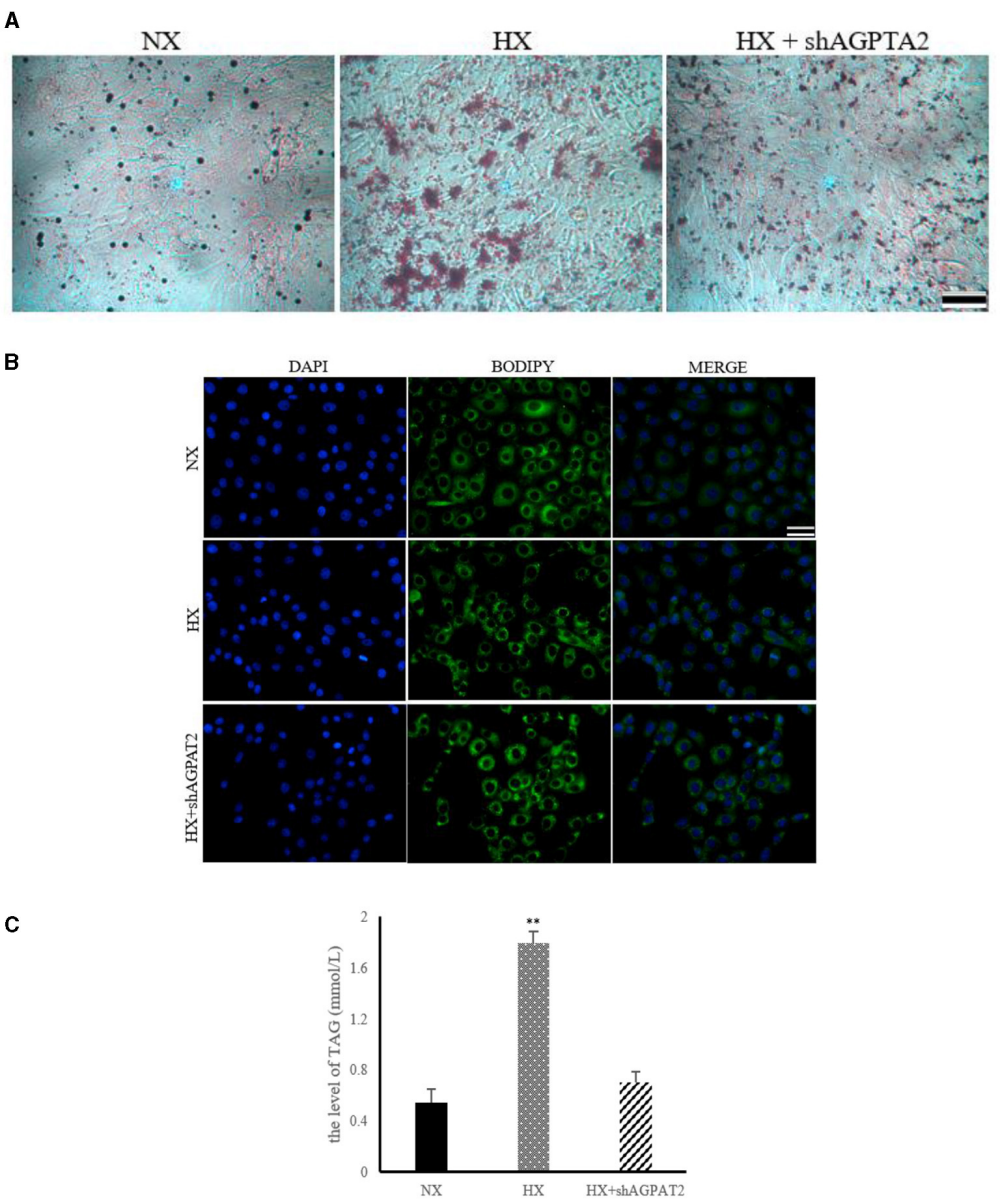
Effect of silencing of AGPAT2 gene on lipid synthesis in BMECs. **(A)** ORO staining of BMECs under silencing of AGPAT2 gene. NX, normoxia; HX, hypoxia; **(B)** BODIPY staining of BMECs under silencing of AGPAT2 gene; **(C)** Level of TAG in BMECs under silencing of AGPAT2 gene; *n* = 3, **p < 0.01.

The effects of glycerophospholipids metabolism on the lipid synthesis induced by hypoxia in BMECs are shown in Figure 7. The results of ORO and BODIPY staining showed significantly reduced (*P* < 0.05) lipid droplet formation in shAGPAT2 group compared with that of hypoxia group, while no significant difference (*P* > 0.05) was shown between control and shAGPAT2 group (Figures 7A,B). The level of TAG in shAGPAT2 group was significantly decreased (*P* < 0.05) than that in hypoxia group, while no significant difference (*P* > 0.05) was found between control and shAGPAT2 group (Figure 7C).

## Discussion

In our previous study on the adaptive mechanism of Holstein dairy cows to hypoxia, we found that the milk fat rate increased obviously. At present, the research on its mechanism is not clear, so we intend to explore it in this paper. The most striking finding of our trial was that milk fat rate levels increased significantly in hypoxia condition. Interestingly, we measured several biochemical indexes related to lipid metabolism, such as cholesterol, high density lipoprotein, ApoA1, and ApoB (32), and found that hypoxia could affect the changes of these indexes in varying degrees, indicating that hypoxia promotes the accumulation of serum lipids, which was consistent with the previous description that TG mobilization and lipid peroxidation could be increased by hypoxia exposure (33).

In this study, a non-target metabolomics approach was used to study the metabolic disorders and adaptive mechanisms associated with hypoxia exposure in dairy cows. The results of metabolomics of plasma showed that glycerophospholipids metabolism was significantly up-regulated (34). As an inflammatory mediator, LysoPC regulated the proliferation and apoptosis of endothelial cells (35). In hypoxic dairy cows, the body adapted to hypoxic stress by regulating inflammation (32), which explained why the level of lysophosphatidic acid (LPA) increased significantly in this experiment.

It was found that the level of free fatty acids (FFA) in plasma was down regulated. Former studies indicate that the level of l-palmitoylcarnitine in plasma can be used as a marker of body FA metabolism. In the present work, we found that the level of l-palmitoylcarnitine was down regulated in plasma metabolomics, which may be due to hypoxia regulating HMG CoA reductase activity to up regulate FA metabolism in hypoxic cows (36).

Phenotypic and metabolomic results of plasma indicate that glycerophospholipid metabolism contributes to hypoxia adaptation. To further investigate whether glycerophospholipid metabolism play an equally important role in mammary epithelial cells, we used metabolomics and lipidomics to verify this.

The results of RT-PCR indicated that hypoxia increased significantly the level of ACACA, FABP3, LPL, PPARG, and SREBP1, which may be due to the up regulation of genes related to FASN and SREBP1 under hypoxia stimulation. SREBP1 regulates FASN, SCD1, and intracellular FABP3 (37, 38). In addition, LPL gene was the key to the uptake and secretion of long-chain fatty acids in milk, and its increased mRNA expression could promote the uptake and transport of long-chain fatty acids, thus promoting milk fat synthesis (39). However, the mRNA levels of CD36 and FASN were significantly decreased, which might be due to that milk fat synthesis was not through the regulation of these genes (40, 41). The results from western blotting indicated that the protein expression of SREBP1 and PPARG increased significantly by hypoxia exposure, which might due to that SREBP1 and PPARG genes were two important factors in regulating mammary milk fat synthesis (42). When migrated from endoplasmic reticulum to Golgi, SREBP1 precursor was hydrolyzed by protease, and then mature SREBP1 with transcriptional activity was released into nucleus by Golgi (43). In conclusion, these results indicated that hypoxia promoted the synthesis of milk fat in BMECs.

In the metabolomics of BMECs, palmitic acid was found to be the marker of fatty acid metabolism, which was consistent with the previous research results (44). The level of palmitic acid was down regulated (FC = 0.73), probably because most of palmitic acid synthesized in mammary gland was used to synthesize triglycerides, leading to the increased level of triglycerides in mammary epithelial cells in this experiment.

Additionally, glycerophospholipids metabolism was up-regulated (34) in BMECs. As an important intermediate in the phospholipid biosynthesis pathway of cell membrane, citicoline was mainly synthesized *in vivo* and is a choline donor (45). Citicoline was mainly composed of two main components, cytidine and choline. In the results of BMECs in this experiment, the level of choline was down regulated, which was consistent with the change of citicoline level. A significant increase in milk fat level was found in the milk of hypoxic cows in this experiment, which may be caused by the increase in the level of glycerol-3-phosphate in BMECs. Studies found that triglycerides were produced by continuous fatty acylation of glycerol-3-phosphate in eukaryotic cells (46), which confirmed the above conjecture. Glycerophosphate ethanolamine was a direct substrate for the synthesis of phosphatidylethanolamine and phosphatidylcholine (47). This experiment found that the increased level of glycerophosphate ethanolamine may be due to the increased synthesis of phosphatidylethanolamine and phosphatidylcholine under hypoxic conditions, thus more glycerophosphate ethanolamine was needed to reshape the body's lipid membrane damage (48).

Lipidomics is a new technology to analyze the final products of lipid metabolism and reveal the internal changes of the whole organism. PCs is the main scaffold of biofilm. A total of 31 PCs increased significantly, including 7 containing polyunsaturated fatty acids (PUFA), 10 containing unsaturated fatty acids, and 13 containing saturated fatty acids. Previous study showed that the fluidity of the membrane was determined by the degree of saturation of the fatty acid chain (49). The increase of PCs containing polyunsaturated fatty acids indicated that the fluidity of cell membrane changes with hypoxia treatment, which promoted the release of lipid produced by mammary epithelial cells into milk (50). PSs was a component of cell membrane. Under normal circumstances, it was maintained in the inner lobule through a family of aminophosphatidylcholine translocase and flipping enzyme (51). In apoptotic cells, PS translocated to the outer lobule, resulting in increased expression. In this experiment, the increase of PS caused the increase of PE, because PS and PE were regulated by the same transport enzyme (52). Exposure of one species to the outer lobule inevitably led to exposure of the other. PG was a precursor of cardiolipin, which was an important component of mitochondrial inner membrane (53). The significant increase of PGs indicated that the structure of mitochondrial membrane was destroyed by hypoxia. TAG was the main component of milk fat. High TAG content might reflect high synthesis rate and low turnover rate (54). The increase of TAGs indicated that hypoxia induced the accumulation of TAG in normal mammary epithelial cells, which reflected the effect of hypoxia on TAG anabolism. In addition, SM is a kind of sphingolipid in cell membrane, and its hydrolysis can produce CER, which is involved in apoptosis signaling pathway (55). SM hydrolysis and CER signaling are essential in the process of apoptosis, which leads to the apoptosis increase. In general, the disorder of lipid level is an important evidence of apoptosis after hypoxia exposure.

Glycerophospholipids metabolism mediated by 1-acylglycero-3-phosphate acyltransferase (AGPAT) plays an important role in the synthesis pathway of TAG (56), which is consistent with our findings in this study. In hypoxic mammary epithelial cells, the silencing of AGPAT2 gene caused reduced intracellular TAG synthesis, possibly because the role of AGPAT2 appeared to be to provide a substrate for the synthesis of glycerophospholipids and TAG in the cell culture model. Overexpression of AGPAT2 in adipocytes increased TAG content (57), which was similar to the results of this study. In addition, gene silencing resulted in down-regulation of the expression of a key protein for TAG synthesis, which was similar to the results of development tests in AGPAT2–/– mice, suggesting that AGPAT2 was critical for glycerophospholipids synthesis in adipose tissue (58). In addition, AGPAT2-induced upregulation of glycerophospholipids metabolism was necessary for LDS enrichment and survival under hypoxia (34).

## Conclusion

In summary, untargeted metabonomics and lipidomics were used in this experiment to study lipid synthesis induced by hypoxia at the metabolic level. The results of metabolomics showed that the metabolism of arginine and proline, glycine, serine and threonine, glycerophospholipids were up-regulated, while the metabolism of fatty acid was down regulated. The results of lipidomics showed that the metabolism of glycerophospholipids was up-regulated by regulating cell apoptosis during hypoxia. In conclusion, we can speculate that Holstein cows adapt to hypoxia exposure mainly by up regulating the glycerophospholipids metabolism. The results of this study are helpful to further understand the mechanism of lipid synthesis related to hypoxia in bovine mammary gland at molecular level.

## Data Availability Statement

The original contributions presented in the study are included in the article/Supplementary Material, further inquiries can be directed to the corresponding author/s.

## Ethics Statement

The animal study was reviewed and approved by Animal Care and Use Guidelines of the Animal Care Committee, Institute of Subtropical Agriculture, Chinese Academy of Sciences, Changsha, China, with protocol ISA-201710.

## Author Contributions

CZ, ZK, QH, and ZT: conceptualization. ZK, BL, and YZ: analysis. ZK: data curation and writing—original draft preparation. CZ and ZT: writing—review and editing. CZ and BL: funding acquisition. All authors have read and approved the final manuscript.

## Funding

The Second Tibetan Plateau Scientific Expedition and Research Program (No. 2019QZKK0501), the Ministry of Science and Technology of China (No. 2018YFD0501903), the Hunan Provincial Science and Technology Department (No. 2017NK1020), the National Natural Science Foundation of China (No. 31772632), and the Youth Innovation Team Project of ISA, Chinese Academy of Sciences (No. 2017QNCXTD_ZCS) supported this work.

## Conflict of Interest

The authors declare that the research was conducted in the absence of any commercial or financial relationships that could be construed as a potential conflict of interest.

## Publisher's Note

All claims expressed in this article are solely those of the authors and do not necessarily represent those of their affiliated organizations, or those of the publisher, the editors and the reviewers. Any product that may be evaluated in this article, or claim that may be made by its manufacturer, is not guaranteed or endorsed by the publisher.
