# Supplementary material for: Multi-Omics Analysis of Mammary Metabolic Changes in Dairy Cows Exposed to Hypoxia
Source: Front Vet Sci. 2021 Oct 14;8:764135. doi: 10.3389/fvets.2021.764135 (PMC8553012; doi:10.3389/fvets.2021.764135)
Supplement: Supplementary file 1 [file Table_1.DOCX]

**Table S1 Summary of differentially expressed metabolites in plasma of dairy cows**

| **MS2 name** | **rt** | **mz** | **VIP** | **P-VALUE** | **FOLD CHANGE** |
| --- | --- | --- | --- | --- | --- |
| Trifluoperazine sulfoxide | 33.773 | 424.167 | 1.2927 | 0.0135 | 1.4720 |
| Choline | 30.544 | 104.107 | 1.7006 | 0.0013 | 3.0603 |
| Acetone oxime | 91.735 | 74.061 | 1.4869 | 0.0020 | 1.5645 |
| Trifluoperazine sulfoxide | 33.773 | 424.167 | 1.2927 | 0.0135 | 1.4720 |
| 17-phenyl trinor Prostaglandin E2 serinol amide | 390.015 | 460.269 | 1.7323 | 0.0144 | 6.4019 |
| 5-AMINOPENTANOATE | 32.015 | 118.087 | 1.4335 | 0.0045 | 1.3994 |
| Creatine | 34.067 | 132.077 | 1.3721 | 0.0065 | 1.4531 |
| Tryptophan | 201.764 | 205.097 | 1.4916 | 0.0012 | 1.9152 |
| Valdecoxib | 34.322 | 315.080 | 1.0734 | 0.0447 | 1.1721 |
| Arginine | 29.789 | 175.119 | 1.5505 | 0.0018 | 2.0700 |
| PC(O-14:1(1E)/0:0) | 413.814 | 452.314 | 1.1214 | 0.0295 | 1.4527 |
| Phe Trp Ser Thr | 240.884 | 540.241 | 1.0712 | 0.0244 | 2.6172 |
| Valine | 52.471 | 118.087 | 1.6032 | 0.0003 | 1.7537 |
| Thiomorpholine 3-carboxylate | 42.204 | 148.043 | 1.5217 | 0.0009 | 1.3367 |
| PC(O-18:2(9Z,12Z)/2:0) | 471.277 | 548.371 | 1.1311 | 0.0318 | 1.6160 |
| (+)-4,11-Eudesmadien-3-one | 329.876 | 219.174 | 1.6456 | 0.0047 | 2.8413 |
| Citrulline | 31.814 | 176.103 | 1.1440 | 0.0198 | 1.5395 |
| 6-Hydroxy-5-methoxyindole glucuronide | 213.716 | 340.103 | 1.4268 | 0.0006 | 12.2411 |
| Urate-3-ribonucleoside | 177.639 | 301.078 | 1.6138 | 0.0379 | 14.1382 |
| 3-ketosphinganine | 409.806 | 300.290 | 1.8022 | 0.0020 | 6.2903 |
| 2-Ethyl-2-methyl-3-hydroxysuccinimide | 202.298 | 158.084 | 1.2812 | 0.0135 | 1.3071 |
| 4-Oxoproline | 30.865 | 130.050 | 1.3047 | 0.0106 | 1.2298 |
| Clofop | 91.444 | 293.054 | 1.2483 | 0.0202 | 1.6253 |
| 7-(1,3-Cyclohexadienyl)-5-hydroxy-2,6-dimethyl-2-hepten-4-one | 455.530 | 235.169 | 1.6620 | 0.0337 | 4.1485 |
| DL-Stearoylcarnitine | 479.233 | 428.373 | 1.6034 | 0.0006 | 2.2600 |
| Alpha-Pyrrolidinopropiophenone | 453.659 | 204.138 | 1.7266 | 0.0292 | 8.1450 |
| PE(18:0/0:0) | 486.915 | 482.324 | 1.1999 | 0.0324 | 1.3122 |
| PC(22:4(7Z,10Z,13Z,16Z)/0:0) | 463.870 | 572.371 | 1.3026 | 0.0088 | 1.9115 |
| dodecanamide | 433.029 | 200.201 | 1.3256 | 0.0129 | 1.4320 |
| (2R,3R,4R)-2-Amino-4-hydroxy-3-methylpentanoic acid | 34.662 | 148.097 | 1.6714 | 0.0001 | 1.4316 |
| 3-Buten-1-amine | 35.400 | 72.081 | 1.7193 | 0.0000 | 1.7802 |
| Palmitoyl-L-carnitine | 448.579 | 400.342 | 1.2991 | 0.0139 | 1.9489 |
| L-gamma-glutamyl-L-valine | 172.269 | 247.129 | 1.3148 | 0.0127 | 1.5817 |
| Methionine | 61.629 | 150.058 | 1.2382 | 0.0087 | 1.6782 |
| Prolyl-Hydroxyproline | 53.621 | 229.118 | 1.0475 | 0.0382 | 1.6048 |
| PC(18:0/0:0) | 485.135 | 524.371 | 1.5705 | 0.0004 | 1.8011 |
| Asperagenin | 444.835 | 449.326 | 1.6384 | 0.0064 | 6.7951 |
| PC(P-15:0/0:0) | 432.565 | 466.329 | 1.5517 | 0.0007 | 1.7036 |
| Serotonin | 167.280 | 177.102 | 1.2568 | 0.0398 | 5.0511 |
| 4-Carboxyphenylglycine | 253.282 | 196.060 | 1.5892 | 0.0005 | 2.0877 |
| 1-(8Z,11Z,14Z-eicosatrienoyl)-sn-glycero-3-phosphocholine | 447.777 | 546.356 | 1.2978 | 0.0153 | 2.1297 |
| phenylacetylglutamine | 226.226 | 265.118 | 1.4782 | 0.0313 | 3.7895 |
| PC(0:0/20:4(5Z,8Z,11Z,14Z)) | 432.179 | 544.340 | 1.1951 | 0.0185 | 1.6157 |
| Scalarin | 479.881 | 445.295 | 1.7771 | 0.0004 | 134.1745 |
| Proline-hydroxyproline | 34.075 | 229.118 | 1.0962 | 0.0116 | 1.7532 |
| C16 Sphingosine | 370.145 | 272.258 | 1.6435 | 0.0086 | 3.0174 |
| PC(18:3(6Z,9Z,12Z)/0:0)[U] | 411.137 | 518.324 | 1.3319 | 0.0129 | 1.7413 |
| Phytosphingosine | 364.544 | 318.300 | 1.4719 | 0.0387 | 3.0121 |
| Lysine | 26.965 | 147.113 | 1.5915 | 0.0001 | 2.2029 |
| 4'-Apo-beta,psi-caroten-4'-al | 462.266 | 483.364 | 1.6908 | 0.0001 | 2.0624 |
| PC(16:0/0:0)[U] | 438.421 | 496.340 | 1.4811 | 0.0005 | 1.6520 |
| PC(15:1(9Z)/0:0) | 409.535 | 480.309 | 1.3656 | 0.0055 | 2.3372 |
| Indole | 202.026 | 118.065 | 1.4959 | 0.0019 | 1.4920 |
| 2-Methylpropanal oxime | 158.686 | 88.076 | 1.4811 | 0.0126 | 1.7004 |
| Elaidic carnitine | 456.356 | 426.358 | 1.1257 | 0.0490 | 1.6248 |
| L-Arginine | 29.003 | 197.101 | 1.5309 | 0.0016 | 1.7836 |
| Sesamex | 268.405 | 299.149 | 1.6897 | 0.0136 | 11.6218 |
| 1,25-dihydroxy-10,19-methano-23-oxavitamin D3 | 482.779 | 433.331 | 1.6922 | 0.0034 | 37.7700 |
| 1,25-Dihydroxy-24-oxo-16-ene-vitamin D3 | 485.536 | 429.300 | 1.2150 | 0.0386 | 3.6629 |
| cis-4-Hydroxy-D-proline | 87.829 | 132.066 | 1.5440 | 0.0129 | 0.3979 |
| piperidine | 34.342 | 86.097 | 1.7445 | 0.0000 | 0.3626 |
| 4-(Trimethylammonio)but-2-enoate | 34.045 | 144.102 | 1.6225 | 0.0205 | 0.1872 |
| 4-Aminomethylcyclohexanecarboxylic acid | 34.126 | 158.118 | 1.5888 | 0.0002 | 0.2871 |
| Pipecolic acid | 34.061 | 130.086 | 1.4347 | 0.0183 | 0.1987 |
| 3-(N-Nitrosomethylamino)propionitrile | 33.517 | 114.067 | 1.0866 | 0.0425 | 0.8338 |
| DL-2-Aminooctanoic acid | 229.663 | 160.133 | 1.5043 | 0.0024 | 0.4366 |
| Dioctyl Phthalate | 591.390 | 391.284 | 1.5169 | 0.0072 | 0.7100 |
| N,N-dimethyl-L-Valine | 192.917 | 146.118 | 1.1593 | 0.0415 | 0.5421 |
| Vigabatrin | 55.185 | 130.086 | 1.3728 | 0.0437 | 0.5319 |
| 3-Indolepropionic acid | 309.251 | 190.086 | 1.2579 | 0.0095 | 0.4729 |
| Methyl acetyl ricinoleate | 521.593 | 355.282 | 1.2094 | 0.0270 | 0.8423 |
| 1-Lauroyl-2-hydroxy-sn-glycero-3-phosphocholine | 367.017 | 440.277 | 1.8005 | 0.0078 | 0.0026 |
| Octadecyl fumarate | 540.856 | 369.297 | 1.3488 | 0.0123 | 0.8039 |
| Bufadienolide | 372.470 | 355.263 | 1.4889 | 0.0275 | 0.2508 |
| Linoleamide | 504.506 | 280.263 | 1.5494 | 0.0009 | 0.7104 |
| D-(+)-Turanose | 31.629 | 365.105 | 1.7527 | 0.0216 | 0.0016 |
| Chol-11-Enic Acid | 372.573 | 373.274 | 1.4832 | 0.0272 | 0.2567 |
| Asn Glu Leu Leu | 374.004 | 488.268 | 1.2668 | 0.0138 | 0.6786 |
| Disulfiram | 450.470 | 297.058 | 1.2244 | 0.0234 | 0.8999 |
| Acutifolane A | 383.963 | 263.164 | 1.2344 | 0.0186 | 0.4932 |
| 7C-aglycone | 280.930 | 299.128 | 1.5954 | 0.0196 | 0.2829 |
| Sphingofungin A | 381.490 | 432.311 | 1.1502 | 0.0261 | 0.5502 |
| Homostachydrine | 207.278 | 158.118 | 1.7001 | 0.0017 | 0.3648 |
| 13S-HpOTrE | 390.010 | 311.222 | 1.7708 | 0.0049 | 0.1175 |
| Betonicine | 183.216 | 160.097 | 1.4537 | 0.0068 | 0.4375 |
| Buprenorphine | 405.256 | 468.309 | 1.2176 | 0.0413 | 0.7004 |
| Simvastatin acid | 475.055 | 437.290 | 1.7413 | 0.0000 | 0.3564 |
| 1-Isothiocyanatobutane | 450.718 | 116.053 | 1.2730 | 0.0166 | 0.9031 |
| N4-Acetylcytidine | 181.540 | 286.103 | 1.5090 | 0.0016 | 0.4456 |
| BENZALKONIUM | 422.911 | 304.300 | 1.8390 | 0.0004 | 0.0473 |
| BISABOLOL ACETATE | 389.747 | 265.216 | 1.7804 | 0.0049 | 0.0951 |
| LysoPE(0:0/22:5(4Z,7Z,10Z,13Z,16Z)) | 438.398 | 528.309 | 1.5152 | 0.0142 | 0.3761 |
| Methyl aminolevulinate | 80.505 | 146.081 | 1.5832 | 0.0191 | 0.1259 |
| LysoPE(20:5(5Z,8Z,11Z,14Z,17Z)/0:0) | 428.524 | 500.275 | 1.2865 | 0.0313 | 0.6875 |
| Cholic Acid | 372.470 | 426.321 | 1.5055 | 0.0126 | 0.3036 |
| N-arachidonoyl alanine | 372.373 | 376.283 | 1.3961 | 0.0335 | 0.2215 |
| Carboxynorspermidine | 30.863 | 176.139 | 1.1838 | 0.0361 | 0.7413 |

**Table S2 Summary of differentially expressed metabolites in BMECs**

| **MS2 name** | **rt** | **mz** | **VIP** | **P-VALUE** | **FOLD CHANGE** |
| --- | --- | --- | --- | --- | --- |
| Trifluoperazine sulfoxide | 33.773 | 424.167 | 1.2468 | 0.01191 | 1.4155 |
| Choline | 30.544 | 104.107 | 1.6712 | 0.00101 | 2.9469 |
| Acetone oxime | 91.735 | 74.061 | 1.4158 | 0.00303 | 1.5103 |
| 17-phenyl trinor Prostaglandin E2 serinol amide | 390.015 | 460.269 | 1.6810 | 0.01602 | 6.1688 |
| 5-AMINOPENTANOATE | 32.015 | 118.087 | 1.2364 | 0.01192 | 1.3523 |
| Creatine | 34.067 | 132.077 | 1.3293 | 0.00665 | 1.3967 |
| Tryptophan | 201.764 | 205.097 | 1.3860 | 0.00364 | 1.8693 |
| Arginine | 29.789 | 175.119 | 1.5109 | 0.00229 | 2.0102 |
| Phe Trp Ser Thr | 240.884 | 540.241 | 1.0264 | 0.02262 | 2.6064 |
| Valine | 52.471 | 118.087 | 1.5086 | 0.00101 | 1.6996 |
| Thiomorpholine 3-carboxylate | 42.204 | 148.043 | 1.2453 | 0.01253 | 1.2939 |
| PC(O-18:2(9Z,12Z)/2:0) | 471.277 | 548.371 | 1.0343 | 0.04250 | 1.5674 |
| (+)-4,11-Eudesmadien-3-one | 329.876 | 219.174 | 1.6232 | 0.00561 | 2.7652 |
| Citrulline | 31.814 | 176.103 | 1.0378 | 0.03744 | 1.4917 |
| 6-Hydroxy-5-methoxyindole glucuronide | 213.716 | 340.103 | 1.3775 | 0.00087 | 11.7952 |
| Urate-3-ribonucleoside | 177.639 | 301.078 | 1.5821 | 0.04007 | 13.4329 |
| 3-ketosphinganine | 409.806 | 300.290 | 1.7496 | 0.00291 | 6.1490 |
| 4-Oxoproline | 30.865 | 130.050 | 1.1125 | 0.03050 | 1.1843 |
| Clofop | 91.444 | 293.054 | 1.2287 | 0.02814 | 1.5770 |
| 7-(1,3-Cyclohexadienyl)-5-hydroxy-2,6-dimethyl-2-hepten-4-one | 455.530 | 235.169 | 1.6084 | 0.03764 | 4.0021 |
| Tambulin 3,5-diacetate | 336.813 | 429.118 | 1.0651 | 0.04692 | 1.0962 |
| DL-Stearoylcarnitine | 479.233 | 428.373 | 1.4637 | 0.00275 | 2.1453 |
| Alpha-Pyrrolidinopropiophenone | 453.659 | 204.138 | 1.6859 | 0.03211 | 7.8849 |
| PC(22:4(7Z,10Z,13Z,16Z)/0:0) | 463.870 | 572.371 | 1.2356 | 0.01086 | 1.8593 |
| dodecanamide | 433.029 | 200.201 | 1.2824 | 0.01196 | 1.3684 |
| (2R,3R,4R)-2-Amino-4-hydroxy-3-methylpentanoic acid | 34.662 | 148.097 | 1.7125 | 0.00001 | 1.3783 |
| 3-Buten-1-amine | 35.400 | 72.081 | 1.6986 | 0.00001 | 1.7265 |
| Palmitoyl-L-carnitine | 448.579 | 400.342 | 1.1979 | 0.02443 | 1.8539 |
| L-gamma-glutamyl-L-valine | 172.269 | 247.129 | 1.2766 | 0.01008 | 1.5261 |
| Methionine | 61.629 | 150.058 | 1.0813 | 0.02212 | 1.6363 |
| PC(18:0/0:0) | 485.135 | 524.371 | 1.4544 | 0.00174 | 1.7367 |
| Asperagenin | 444.835 | 449.326 | 1.5858 | 0.00740 | 6.7792 |
| PC(P-15:0/0:0) | 432.565 | 466.329 | 1.4434 | 0.00192 | 1.6664 |
| 4-Carboxyphenylglycine | 253.282 | 196.060 | 1.5182 | 0.00084 | 2.0075 |
| 1-(8Z,11Z,14Z-eicosatrienoyl)-sn-glycero-3-phosphocholine | 447.777 | 546.356 | 1.1981 | 0.02062 | 2.0562 |
| phenylacetylglutamine | 226.226 | 265.118 | 1.3744 | 0.03950 | 3.6871 |
| PC(0:0/20:4(5Z,8Z,11Z,14Z)) | 432.179 | 544.340 | 1.0954 | 0.02828 | 1.5576 |
| Scalarin | 479.881 | 445.295 | 1.7292 | 0.00076 | 129.2229 |
| Proline-hydroxyproline | 34.075 | 229.118 | 1.0362 | 0.02029 | 1.7133 |
| C16 Sphingosine | 370.145 | 272.258 | 1.5533 | 0.01104 | 2.9519 |
| PC(18:3(6Z,9Z,12Z)/0:0)[U] | 411.137 | 518.324 | 1.2309 | 0.01911 | 1.6901 |
| Phytosphingosine | 364.544 | 318.300 | 1.4310 | 0.02954 | 2.7934 |
| Lysine | 26.965 | 147.113 | 1.5526 | 0.00019 | 2.1524 |
| 4'-Apo-beta,psi-caroten-4'-al | 462.266 | 483.364 | 1.6256 | 0.00016 | 1.9998 |
| PC(16:0/0:0)[U] | 438.421 | 496.340 | 1.3985 | 0.00138 | 1.6012 |
| PC(15:1(9Z)/0:0) | 409.535 | 480.309 | 1.2822 | 0.00986 | 2.2982 |
| Indole | 202.026 | 118.065 | 1.3356 | 0.00920 | 1.4541 |
| 2-Methylpropanal oxime | 158.686 | 88.076 | 1.4256 | 0.01875 | 1.6476 |
| Octylamine | 221.719 | 130.159 | 1.3077 | 0.00847 | 1.0838 |
| L-Arginine | 29.003 | 197.101 | 1.4847 | 0.00224 | 1.7291 |
| Sesamex | 268.405 | 299.149 | 1.6443 | 0.01388 | 11.0661 |
| 1,25-dihydroxy-10,19-methano-23-oxavitamin D3 | 482.779 | 433.331 | 1.6341 | 0.00329 | 35.1870 |
| 1,25-Dihydroxy-24-oxo-16-ene-vitamin D3 | 485.536 | 429.300 | 1.1119 | 0.04235 | 3.5078 |
| Bufadienolide | 372.470 | 355.263 | 1.4673 | 0.02212 | 0.2428 |
| 2,5-Furandicarboxylic acid | 24.991 | 157.015 | 1.2215 | 0.02015 | 0.7877 |
| Linoleamide | 504.506 | 280.263 | 1.5328 | 0.00121 | 0.6807 |
| D-(+)-Turanose | 31.629 | 365.105 | 1.7043 | 0.01866 | 0.0016 |
| Theaspirone A | 455.282 | 209.154 | 1.2371 | 0.01611 | 0.9406 |
| Chol-11-Enic Acid | 372.573 | 373.274 | 1.4627 | 0.02180 | 0.2483 |
| Methyl carbamate | 682.481 | 76.040 | 1.1471 | 0.03042 | 0.7250 |
| Asn Glu Leu Leu | 374.004 | 488.268 | 1.2527 | 0.01039 | 0.6576 |
| 1-Butylamine | 220.474 | 74.097 | 1.4405 | 0.00248 | 0.9108 |
| cis-4-Hydroxy-D-proline | 87.829 | 132.066 | 1.5575 | 0.01100 | 0.3816 |
| piperidine | 34.342 | 86.097 | 1.7096 | 0.00002 | 0.3470 |
| 4-(Trimethylammonio)but-2-enoate | 34.045 | 144.102 | 1.6010 | 0.01775 | 0.1832 |
| Cyclohexylamine | 162.947 | 100.112 | 1.1761 | 0.02327 | 0.9614 |
| 4-Aminomethylcyclohexanecarboxylic acid | 34.126 | 158.118 | 1.5364 | 0.00017 | 0.2874 |
| Pipecolic acid | 34.061 | 130.086 | 1.4157 | 0.01643 | 0.1929 |
| 3-(N-Nitrosomethylamino)propionitrile | 33.517 | 114.067 | 1.2108 | 0.01646 | 0.8086 |
| DL-2-Aminooctanoic acid | 229.663 | 160.133 | 1.5395 | 0.00135 | 0.4066 |
| Dioctyl Phthalate | 591.390 | 391.284 | 1.4652 | 0.01044 | 0.6815 |
| N,N-dimethyl-L-Valine | 192.917 | 146.118 | 1.2361 | 0.01527 | 0.5069 |
| Vigabatrin | 55.185 | 130.086 | 1.3859 | 0.03753 | 0.5137 |
| 3-Indolepropionic acid | 309.251 | 190.086 | 1.3131 | 0.00504 | 0.4563 |
| Methyl acetyl ricinoleate | 521.593 | 355.282 | 1.2242 | 0.02638 | 0.8074 |
| Difenoconazole | 440.554 | 406.072 | 1.0665 | 0.04833 | 0.9059 |
| Phthalic anhydride | 437.880 | 149.023 | 1.3532 | 0.00661 | 0.9088 |
| 1-Lauroyl-2-hydroxy-sn-glycero-3-phosphocholine | 367.017 | 440.277 | 1.7534 | 0.00781 | 0.0025 |
| Octadecyl fumarate | 540.856 | 369.297 | 1.2276 | 0.04770 | 0.7683 |
| Disulfiram | 450.470 | 297.058 | 1.3489 | 0.01902 | 0.8644 |
| Acutifolane A | 383.963 | 263.164 | 1.1725 | 0.01870 | 0.4784 |
| 7C-aglycone | 280.930 | 299.128 | 1.5483 | 0.01916 | 0.2733 |
| Sphingofungin A | 381.490 | 432.311 | 1.1160 | 0.02041 | 0.5527 |
| Homostachydrine | 207.278 | 158.118 | 1.6737 | 0.00496 | 0.3433 |
| 13S-HpOTrE | 390.010 | 311.222 | 1.7358 | 0.00449 | 0.1142 |
| Betonicine | 183.216 | 160.097 | 1.4152 | 0.01701 | 0.4152 |
| Buprenorphine | 405.256 | 468.309 | 1.2055 | 0.02065 | 0.6796 |
| Simvastatin acid | 475.055 | 437.290 | 1.6640 | 0.00002 | 0.3464 |
| 1-Isothiocyanatobutane | 450.718 | 116.053 | 1.1881 | 0.02576 | 0.8694 |
| N4-Acetylcytidine | 181.540 | 286.103 | 1.5447 | 0.00020 | 0.4252 |
| BENZALKONIUM | 422.911 | 304.300 | 1.7956 | 0.00043 | 0.0451 |
| 7-Ketodeoxycholic acid | 500.635 | 424.363 | 1.4114 | 0.01165 | 0.8929 |
| BISABOLOL ACETATE | 389.747 | 265.216 | 1.7434 | 0.00456 | 0.0926 |
| LysoPC(22:5(4Z,7Z,10Z,13Z,16Z)) | 430.795 | 570.347 | 1.1304 | 0.03915 | 0.6894 |
| LysoPE(0:0/22:5(4Z,7Z,10Z,13Z,16Z)) | 438.398 | 528.309 | 1.4806 | 0.01005 | 0.3654 |
| Methyl aminolevulinate | 80.505 | 146.081 | 1.5428 | 0.02132 | 0.1205 |
| LysoPE(20:5(5Z,8Z,11Z,14Z,17Z)/0:0) | 428.524 | 500.275 | 1.2830 | 0.02499 | 0.6620 |
| Tiabendazole | 202.710 | 202.044 | 1.1517 | 0.04079 | 0.9890 |
| Cholic Acid | 372.470 | 426.321 | 1.4837 | 0.00950 | 0.2930 |
| N-arachidonoyl alanine | 372.373 | 376.283 | 1.3718 | 0.02772 | 0.2138 |
| Carboxynorspermidine | 30.863 | 176.139 | 1.3406 | 0.01057 | 0.7148 |

**Table S3 Summary of differentially expressed metabolites in BMECs**

| **MS2 name** | **rt** | **mz** | **VIP** | **P-value** | **Fold Change** |
| --- | --- | --- | --- | --- | --- |
| Deoxyinosine | 187.715 | 253.093 | 1.3728 | 0.00041 | 1.8191 |
| Cytidine monophosphate | 450.412 | 324.059 | 1.4341 | 0.00018 | 3.7601 |
| Thymine | 87.300 | 127.050 | 1.2686 | 0.00672 | 2.3778 |
| 5-Methyldeoxycytidine | 208.787 | 242.114 | 1.3860 | 0.00711 | 2.3225 |
| Deoxyadenosine | 139.928 | 252.109 | 1.0601 | 0.04524 | 1.6598 |
| (E)-Antibiotic BE 23372M | 368.588 | 313.074 | 1.0430 | 0.00088 | 3.2477 |
| N,N-Dimethylsphingosine | 69.905 | 328.320 | 1.0842 | 0.00193 | 3.6196 |
| 1-Methyladenosine | 124.110 | 282.120 | 1.1224 | 0.03306 | 1.6348 |
| Anserine | 427.030 | 241.130 | 1.1357 | 0.00965 | 1.9496 |
| dCMP | 452.957 | 308.064 | 1.4583 | 0.00007 | 2.4131 |
| Xanthine | 223.776 | 153.040 | 1.4743 | 0.00479 | 3.5159 |
| Endoxifen | 460.117 | 374.215 | 1.4667 | 0.00008 | 2.9551 |
| Glycerol 3-phosphate | 406.588 | 173.021 | 1.0052 | 0.02240 | 1.5538 |
| Digoxin | 413.526 | 803.416 | 1.3511 | 0.02254 | 4.0081 |
| Nummularine B | 393.472 | 592.320 | 1.4010 | 0.00001 | 10.9182 |
| Morellin | 481.782 | 545.257 | 1.4581 | 0.00352 | 13.6863 |
| LysoPE(0:0/20:2(11Z,14Z)) | 208.888 | 506.324 | 1.1920 | 0.00645 | 1.7272 |
| Galactosylsphingosine | 132.405 | 462.344 | 1.1815 | 0.00274 | 2.0575 |
| Citreoviridinol A1 | 425.177 | 421.187 | 1.4495 | 0.01843 | 7.9750 |
| AICA-riboside | 208.728 | 259.103 | 1.0833 | 0.01926 | 2.6284 |
| N-Succinyl-2-amino-6-ketopimelate | 244.990 | 290.086 | 1.3861 | 0.00174 | 1.5478 |
| Lactosylceramide (d18:1/16:0) | 204.344 | 862.626 | 1.2100 | 0.00591 | 1.5091 |
| Threoninyl-Tyrosine | 297.088 | 283.123 | 1.1574 | 0.01236 | 1.4538 |
| Arachidyl carnitine | 190.376 | 456.405 | 1.2231 | 0.02696 | 2.4665 |
| Benzoyl ecgonine | 138.227 | 290.141 | 1.1145 | 0.01518 | 1.6015 |
| Kanzonol V | 464.442 | 377.178 | 1.3293 | 0.00803 | 6.1059 |
| Cinncassiol D2 glucoside | 467.701 | 531.278 | 1.3110 | 0.01641 | 7.1413 |
| Sedoheptulose 1-phosphate | 187.790 | 291.049 | 1.4194 | 0.00023 | 2.3462 |
| Aspartyl-Arginine | 455.712 | 290.145 | 1.4567 | 0.00005 | 4.4838 |
| trans-Aconitic acid | 223.109 | 175.023 | 1.1570 | 0.00453 | 1.8003 |
| Isoleucyl-Lysine | 286.036 | 260.197 | 1.3932 | 0.01965 | 7.7240 |
| Aspartyl-Asparagine | 439.610 | 248.088 | 1.1425 | 0.01085 | 1.6975 |
| 2-S-glutathionyl acetate | 464.327 | 366.097 | 1.0336 | 0.04249 | 1.6810 |
| gamma-Glutamylglutamic acid | 473.855 | 277.103 | 1.3175 | 0.01324 | 1.8925 |
| Melezitose | 188.039 | 543.135 | 1.2849 | 0.03217 | 71.4435 |
| PE(P-16:0/16:1(9Z)) | 86.833 | 674.513 | 1.2490 | 0.00414 | 3.0244 |
| N-Acetyl-glucosamine 1-phosphate | 464.429 | 324.046 | 1.5862 | 0.00053 | 124.6840 |
| Glycerylphosphorylethanolamine | 406.590 | 216.063 | 1.0372 | 0.04075 | 1.3339 |
| PE(P-18:1(11Z)/14:0) | 162.529 | 674.512 | 1.3052 | 0.00248 | 2.4334 |
| Adenine | 300.532 | 136.062 | 1.5253 | 0.00209 | 0.3075 |
| L-Proline | 403.630 | 116.071 | 1.1615 | 0.01427 | 0.5626 |
| Ornithine | 531.285 | 133.097 | 1.4515 | 0.00005 | 0.3710 |
| L-Isoleucine | 288.777 | 132.102 | 1.1877 | 0.00696 | 0.7366 |
| Choline | 281.757 | 104.107 | 1.5242 | 0.00000 | 0.4159 |
| beta-Alanine | 357.322 | 90.055 | 1.5100 | 0.00000 | 0.3787 |
| Cytosine | 251.178 | 112.051 | 1.4566 | 0.00007 | 0.6128 |
| Inosine | 227.234 | 269.088 | 1.5011 | 0.00001 | 0.4844 |
| Creatinine | 174.573 | 114.066 | 1.4178 | 0.00028 | 0.4768 |
| Cytidine | 251.308 | 244.093 | 1.4386 | 0.00007 | 0.5938 |
| L-Glutamic acid | 448.999 | 148.060 | 1.4283 | 0.00134 | 0.4331 |
| Niacinamide | 57.846 | 123.055 | 1.3578 | 0.00105 | 0.6661 |
| L-Carnitine | 367.391 | 162.112 | 1.3096 | 0.00174 | 0.5392 |
| Piperidine | 277.913 | 86.097 | 1.1072 | 0.01427 | 0.7567 |
| Deoxyguanosine | 176.288 | 268.104 | 1.3974 | 0.00007 | 0.4130 |
| Zymonic acid | 174.563 | 159.027 | 1.2111 | 0.00459 | 0.7538 |
| L-Serine | 388.114 | 106.050 | 1.4026 | 0.00025 | 0.5647 |
| 1-deoxy-1-(N6-lysino)-D-fructose | 416.359 | 134.045 | 1.4549 | 0.00011 | 0.4149 |
| gamma-Aminobutyric acid | 384.805 | 104.071 | 1.3019 | 0.00459 | 0.6628 |
| Riboflavin | 228.867 | 377.145 | 1.3538 | 0.00042 | 0.5191 |
| Protein serine | 429.638 | 106.050 | 1.2947 | 0.00150 | 0.6818 |
| Diethanolamine | 312.043 | 106.086 | 1.1858 | 0.00964 | 0.6471 |
| 3-Dehydroxycarnitine | 390.586 | 146.117 | 1.3321 | 0.00059 | 0.6387 |
| Ethenyl acetate | 384.667 | 87.044 | 1.3205 | 0.00420 | 0.6610 |
| Creatine | 370.333 | 132.077 | 1.3707 | 0.01713 | 0.3708 |
| Metenamine | 267.994 | 141.113 | 1.4585 | 0.00007 | 0.6520 |
| N2,N2-Dimethylguanosine | 203.624 | 312.130 | 1.0631 | 0.03244 | 0.6135 |
| 1,2,3,4-Tetrahydro-b-carboline-1,3-dicarboxylic acid | 318.140 | 261.087 | 1.3164 | 0.00130 | 0.6740 |
| L-Methionine | 297.891 | 150.058 | 1.3322 | 0.00100 | 0.6247 |
| Citrulline | 403.959 | 176.103 | 1.3869 | 0.00013 | 0.5805 |
| L-Asparagine | 388.248 | 133.061 | 1.1985 | 0.00513 | 0.7102 |
| Flavidulol B | 30.947 | 259.169 | 1.1322 | 0.01213 | 0.7798 |
| Pyrrolidonecarboxylic acid | 410.090 | 130.050 | 1.4821 | 0.00001 | 0.4671 |
| Guanosine | 277.315 | 284.098 | 1.0037 | 0.01843 | 0.7738 |
| Isoleucyl-Leucine | 202.614 | 245.186 | 1.1752 | 0.03113 | 0.3571 |
| Leucyl-Aspartate | 391.517 | 247.129 | 1.3299 | 0.00125 | 0.5065 |
| Inosinic acid | 459.839 | 349.054 | 1.4309 | 0.00003 | 0.3250 |
| Guanosine monophosphate | 476.178 | 364.065 | 1.3287 | 0.00031 | 0.5381 |
| Dimethylglycine | 319.875 | 104.071 | 1.5002 | 0.00228 | 0.2160 |
| Uridine 5'-monophosphate | 453.544 | 325.042 | 1.5253 | 0.00000 | 0.3510 |
| 2-Aminoacrylic acid | 416.328 | 88.040 | 1.4488 | 0.00007 | 0.3808 |
| 3-Aminocaproic acid | 398.721 | 132.102 | 1.2274 | 0.00465 | 0.3856 |
| L-alpha-Aminobutyric acid | 336.644 | 104.071 | 1.0118 | 0.02776 | 0.6875 |
| Alanyl-Leucine | 247.545 | 203.139 | 1.1078 | 0.00947 | 0.5009 |
| O-Demethylforbexanthone | 370.389 | 327.090 | 1.0888 | 0.02935 | 0.4253 |
| N-Methyl-a-aminoisobutyric acid | 396.995 | 118.086 | 1.1032 | 0.02169 | 0.6260 |
| Mizolastine | 353.532 | 455.203 | 1.1728 | 0.00109 | 0.2745 |
| D-Alanyl-D-alanine | 82.979 | 161.092 | 1.4818 | 0.00001 | 0.4768 |
| Guanosine diphosphate | 473.158 | 444.031 | 1.2528 | 0.02881 | 0.2128 |
| Glycyl-Valine | 299.143 | 175.108 | 1.0361 | 0.02819 | 0.5585 |
| Phytosphingosine | 57.854 | 318.299 | 1.1183 | 0.01270 | 0.7318 |
| Lauroyl diethanolamide | 5.168 | 288.253 | 1.4719 | 0.00002 | 0.6635 |
| Adenosine 2'-phosphate | 442.563 | 348.070 | 1.5282 | 0.00018 | 0.2323 |
| 2-(3,4-Dihydroxybenzoyloxy)-4,6-dihydroxybenzoate | 226.510 | 307.043 | 1.4518 | 0.00005 | 0.6163 |
| L-Glutamine | 385.555 | 147.076 | 1.5677 | 0.00001 | 0.1778 |
| N-Acetylputrescine | 318.200 | 131.118 | 1.0344 | 0.01966 | 0.7983 |
| 6-Methylquinoline | 211.354 | 144.081 | 1.0489 | 0.00936 | 0.4896 |
| 2,5-Dihydro-2,4-dimethyloxazole | 343.771 | 100.076 | 1.4372 | 0.00191 | 0.5130 |
| Methionine sulfoxide | 380.091 | 166.053 | 1.3863 | 0.00021 | 0.5524 |
| Isoleucyl-Threonine | 249.214 | 233.150 | 1.1970 | 0.01150 | 0.4886 |
| 5-Aminoimidazole ribonucleotide | 271.606 | 296.064 | 1.3204 | 0.02403 | 0.0790 |
| 3-Amino-2-piperidone | 514.286 | 115.087 | 1.3948 | 0.00024 | 0.5124 |
| Pyridoxal | 80.387 | 168.065 | 1.1411 | 0.01053 | 0.6668 |
| 5-Aminopentanal | 206.217 | 102.092 | 1.3284 | 0.00028 | 0.6666 |
| Pyroglutamic acid | 385.578 | 130.050 | 1.5672 | 0.00001 | 0.1823 |
| L-Acetylcarnitine | 319.879 | 204.123 | 1.0187 | 0.03197 | 0.6527 |
| Carnosine | 411.830 | 227.103 | 1.0008 | 0.03103 | 0.6151 |
| D-Alanine | 415.303 | 90.055 | 1.4892 | 0.00001 | 0.5439 |
| Testosterone sulfate | 321.624 | 369.169 | 1.3724 | 0.00058 | 0.4914 |
| Styrene | 34.468 | 105.070 | 1.1529 | 0.01276 | 0.6260 |
| N-a-Acetyl-L-arginine | 382.903 | 217.129 | 1.4438 | 0.00003 | 0.4270 |
| Butyrylcarnitine | 274.363 | 232.154 | 1.4992 | 0.00060 | 0.2001 |
| L-trans-4-Methyl-2-pyrrolidinecarboxylic acid | 60.427 | 130.086 | 1.0773 | 0.01258 | 0.6746 |
| L-Aspartic acid | 359.042 | 134.019 | 1.1841 | 0.01259 | 0.5635 |
| Phenylalanyl-Alanine | 223.786 | 237.123 | 1.0093 | 0.01210 | 0.5920 |
| 2-Pyrrolidinone | 384.721 | 86.060 | 1.2984 | 0.00508 | 0.6705 |
| Dethiobiotin | 300.732 | 215.139 | 1.3007 | 0.00284 | 0.5302 |
| LysoPE(0:0/22:5(4Z,7Z,10Z,13Z,16Z)) | 209.672 | 528.309 | 1.2491 | 0.00263 | 0.6591 |
| Polyoxyethylene dioleate | 214.373 | 591.536 | 1.2587 | 0.00479 | 0.3952 |
| Leucyl-Methionine | 204.413 | 263.142 | 1.0844 | 0.03209 | 0.3335 |
| (Â±)-2-Methylthiazolidine | 297.913 | 104.053 | 1.4770 | 0.00006 | 0.2790 |
| 6-Deoxyfagomine | 349.219 | 132.102 | 1.3967 | 0.00044 | 0.6240 |
| 6-Epi-7-isocucurbic acid glucoside | 348.175 | 375.198 | 1.4579 | 0.00004 | 0.6529 |
| Alanyl-Tyrosine | 25.089 | 253.118 | 1.2569 | 0.01504 | 0.5783 |
| Prolyl-Arginine | 421.606 | 272.172 | 1.1209 | 0.01069 | 0.6987 |
| Schizotenuin F | 478.138 | 553.138 | 1.2459 | 0.00367 | 0.6686 |
| Dihydroroseoside | 391.470 | 389.215 | 1.4537 | 0.00009 | 0.3514 |
| Leucyl-Valine | 213.176 | 231.170 | 1.0422 | 0.03412 | 0.3697 |
| Phenylalanylproline | 254.169 | 263.139 | 1.2167 | 0.00267 | 0.6432 |
| Arginyl-Phenylalanine | 354.207 | 322.187 | 1.3737 | 0.00241 | 0.6671 |
| Menthone 1,2-glyceryl ketal | 431.386 | 298.100 | 1.3254 | 0.00010 | 0.6194 |
| LysoPE(20:4(8Z,11Z,14Z,17Z)/0:0) | 421.514 | 502.298 | 1.4579 | 0.00004 | 0.6529 |
| 1,3-Diisopropylbenzene | 31.883 | 163.148 | 1.1281 | 0.01605 | 0.6190 |
| Adenosine 3',5'-diphosphate | 472.610 | 428.036 | 1.5244 | 0.00061 | 0.2738 |
| Propionylcarnitine | 295.322 | 218.138 | 1.1245 | 0.00655 | 0.6349 |
| S-Adenosylmethionine | 532.129 | 399.144 | 1.1802 | 0.00724 | 0.6679 |
| 7-Formyldehydrothalicsimidine | 369.459 | 412.180 | 1.4579 | 0.00004 | 0.6529 |
| S-Adenosylhomocysteine | 396.117 | 385.129 | 1.4833 | 0.00014 | 0.4028 |
| m-Xylene | 33.599 | 107.086 | 1.0834 | 0.02275 | 0.8709 |
| 5-Aminopentanamide | 343.187 | 117.102 | 1.4591 | 0.00141 | 0.4683 |
| 1,2-Dihydro-1,1,6-trimethylnaphthalene | 31.868 | 173.132 | 1.0357 | 0.03111 | 0.6364 |
| 6'-Apiosyllotaustralin | 346.751 | 394.170 | 1.3250 | 0.00699 | 0.4705 |
| Serylvaline | 303.054 | 205.118 | 1.1531 | 0.02928 | 0.4918 |
| Ephedrannin A | 375.731 | 557.109 | 1.1798 | 0.00692 | 0.6860 |
| Valyl-Tyrosine | 239.722 | 281.149 | 1.2692 | 0.00541 | 0.5157 |
| (alpha-D-mannosyl)7-beta-D-mannosyl-diacetylchitobiosyl-L-asparagine, isoform A (protein) | 49.178 | 90.055 | 1.2749 | 0.02384 | 0.4046 |
| N2-gamma-Glutamylglutamine | 443.552 | 276.119 | 1.1966 | 0.00619 | 0.6148 |
| Isoleucyl-Phenylalanine | 192.983 | 279.170 | 1.0355 | 0.02766 | 0.3472 |
| 2'-Oxoaloesol 7-glucoside | 299.643 | 395.134 | 1.0135 | 0.04837 | 0.1575 |
| Mangiferdesmethylursanone | 30.942 | 429.373 | 1.2303 | 0.00202 | 0.6328 |
| Citicoline | 456.548 | 489.114 | 1.0195 | 0.04261 | 0.7256 |
| Phenylalanyl-Valine | 199.623 | 265.154 | 1.3657 | 0.01173 | 0.3847 |
| Valyl-Valine | 228.805 | 217.155 | 1.0398 | 0.01068 | 0.4189 |
| Aspartyl-Glutamate | 471.883 | 263.087 | 1.2814 | 0.00267 | 0.6454 |
| 4-Methylbenzaldehyde | 34.475 | 121.065 | 1.1765 | 0.01026 | 0.5633 |
| Ampicillin | 208.804 | 350.163 | 1.4198 | 0.00445 | 0.3000 |
| Montecristin | 30.066 | 575.503 | 1.4812 | 0.00008 | 0.4721 |
| Isotheaflavin | 443.260 | 565.138 | 1.3647 | 0.00097 | 0.6943 |
| dTDP-4-acetamido-4,6-dideoxy-D-galactose | 457.749 | 590.120 | 1.3705 | 0.00045 | 0.7051 |
| 7-Ketocholesterol | 31.868 | 401.341 | 1.5569 | 0.00000 | 0.3463 |
| Cohibin B | 215.811 | 577.518 | 1.1545 | 0.00709 | 0.7374 |
| PI(18:2(9Z,12Z)/18:2(9Z,12Z)) | 213.079 | 859.532 | 1.1108 | 0.01197 | 0.5466 |
| Ipriflavone | 214.117 | 281.116 | 1.2877 | 0.00980 | 0.4903 |
| Glutamylthreonine | 429.489 | 249.108 | 1.5009 | 0.00440 | 0.0712 |
| Serylserine | 366.904 | 193.082 | 1.2292 | 0.00231 | 0.3759 |
| PC(22:4(7Z,10Z,13Z,16Z)/16:0) | 153.947 | 810.600 | 1.4256 | 0.00014 | 0.5136 |
| Galactopinitol B | 374.844 | 357.138 | 1.4579 | 0.00004 | 0.6529 |
| LysoPC(18:4(6Z,9Z,12Z,15Z)) | 432.231 | 516.315 | 1.4579 | 0.00004 | 0.6529 |
| L-2-Amino-3-methylenehexanoic acid | 371.626 | 144.102 | 1.3205 | 0.00222 | 0.5221 |
| 3-Methyl-5-pentyl-2-furanundecanoic acid | 261.022 | 337.274 | 1.0808 | 0.03000 | 0.5341 |
| SM(d18:1/16:0) | 201.770 | 703.573 | 1.3944 | 0.00005 | 0.6336 |
| 1,2-Dihydroxy-3-keto-5-methylthiopentene | 121.519 | 163.042 | 1.4515 | 0.00029 | 0.5424 |
| PI(20:3(5Z,8Z,11Z)/20:3(5Z,8Z,11Z)) | 209.791 | 911.562 | 1.1510 | 0.00354 | 0.3365 |
| 1-Methyl 2-galloylgalactarate | 434.130 | 377.074 | 1.1907 | 0.00693 | 0.7076 |
| Homocitrulline | 433.201 | 190.119 | 1.4410 | 0.00145 | 0.4503 |
| PC(22:6(4Z,7Z,10Z,13Z,16Z,19Z)/18:0) | 38.776 | 834.601 | 1.4305 | 0.00008 | 0.4903 |
| Acetylcholine | 189.349 | 146.117 | 1.1314 | 0.03336 | 0.0237 |
| Succinyladenosine | 420.634 | 384.114 | 1.1407 | 0.01553 | 0.5269 |
| Diethylcarbamazine N-oxide | 353.208 | 216.170 | 1.3080 | 0.00605 | 0.5842 |
| 4-Methoxybenzyl propanoate | 401.323 | 195.101 | 1.5011 | 0.00001 | 0.5986 |
| Dieporeticenin | 215.891 | 573.489 | 1.2465 | 0.00671 | 0.4058 |
| Tyrosine methylester | 45.258 | 196.097 | 1.1967 | 0.00884 | 0.5538 |
| PE(18:3(6Z,9Z,12Z)/15:0) | 169.414 | 700.491 | 1.4028 | 0.00027 | 0.4122 |
| Phosphocreatine | 456.438 | 212.043 | 1.5763 | 0.00042 | 0.2005 |
| PC(20:2(11Z,14Z)/15:0) | 62.124 | 772.582 | 1.4547 | 0.00023 | 0.3658 |
| FAD | 411.816 | 786.163 | 1.1032 | 0.00749 | 0.6561 |
| Glutaminyllysine | 460.241 | 275.171 | 1.0099 | 0.02746 | 0.6861 |
| Ophthalmic acid | 411.825 | 290.135 | 1.1550 | 0.00904 | 0.5429 |
| Hydroxyprolyl-Tyrosine | 229.881 | 295.128 | 1.0702 | 0.01896 | 0.6358 |
| Blumenol C glucoside | 439.024 | 373.220 | 1.3759 | 0.01106 | 0.2452 |
| Linoelaidyl carnitine | 195.549 | 424.342 | 1.1528 | 0.02078 | 0.2738 |
| Persicaxanthin | 209.598 | 385.273 | 1.3951 | 0.00283 | 0.5014 |
| Cholesta-4,6-dien-3-one | 31.891 | 383.331 | 1.5373 | 0.00107 | 0.2758 |
| PC(22:6(4Z,7Z,10Z,13Z,16Z,19Z)/22:2(13Z,16Z)) | 146.897 | 886.630 | 1.3251 | 0.00316 | 0.2318 |
| Valyl-Methionine | 216.797 | 249.127 | 1.0599 | 0.03524 | 0.2629 |
| 1,2,3,4,Tetrahydro-1,5,7-trimethylnapthalene | 31.870 | 175.148 | 1.2106 | 0.00365 | 0.5575 |
| DL-Glutamate | 526.798 | 148.060 | 1.4565 | 0.00004 | 0.4740 |
| 29-Norcycloartane-3,24-dione | 31.397 | 427.357 | 1.0531 | 0.03759 | 0.6721 |
| Theogallin | 432.287 | 345.084 | 1.3303 | 0.00074 | 0.6877 |
| PC(22:5(7Z,10Z,13Z,16Z,19Z)/16:1(9Z)) | 154.728 | 806.571 | 1.4841 | 0.00013 | 0.4665 |
| PC(P-16:0/20:3(5Z,8Z,11Z)) | 155.055 | 768.590 | 1.0960 | 0.01829 | 0.6736 |
| PC(20:4(8Z,11Z,14Z,17Z)/15:0) | 120.135 | 768.551 | 1.2989 | 0.00903 | 0.4530 |
| L-prolyl-L-proline | 427.802 | 213.123 | 1.3718 | 0.00051 | 0.4427 |
| (5alpha,8beta,9beta)-5,9-Epoxy-3,6-megastigmadien-8-ol | 32.712 | 209.154 | 1.1088 | 0.02069 | 0.6476 |
| 2-[4-(3-Hydroxypropyl)-2-methoxyphenoxy]-1,3-propanediol 1-xyloside | 429.750 | 389.178 | 1.4433 | 0.00003 | 0.4512 |
| 5'-Deoxy-5'-(methylsulfinyl)adenosine | 234.983 | 314.091 | 1.5416 | 0.00017 | 0.4810 |
| PC(20:3(8Z,11Z,14Z)/P-18:0) | 153.059 | 796.622 | 1.1708 | 0.00955 | 0.6895 |
| Glutamylalanine | 424.366 | 219.097 | 1.3485 | 0.00196 | 0.2673 |
| 4-trans-Hydroxyglipizide | 479.867 | 462.183 | 1.4579 | 0.00004 | 0.6529 |
| PS(14:1(9Z)/14:1(9Z)) | 236.944 | 676.418 | 1.3053 | 0.00308 | 0.4239 |
| PC(22:4(7Z,10Z,13Z,16Z)/22:1(13Z)) | 147.862 | 892.677 | 1.3572 | 0.00111 | 0.4722 |
| PC(20:3(8Z,11Z,14Z)/15:0) | 162.506 | 770.568 | 1.0640 | 0.03817 | 0.5518 |
| Demethylated antipyrine | 133.882 | 175.087 | 1.4101 | 0.00016 | 0.5518 |
| PC(22:1(13Z)/14:1(9Z)) | 158.216 | 786.599 | 1.5278 | 0.00000 | 0.6070 |
| Formiminoglutamic acid | 413.646 | 175.071 | 1.4821 | 0.00001 | 0.3872 |
| L-cis-3-Amino-2-pyrrolidinecarboxylic acid | 357.365 | 131.082 | 1.4962 | 0.00000 | 0.3505 |
| PS(18:0/20:4(5Z,8Z,11Z,14Z)) | 213.069 | 812.544 | 1.3343 | 0.00190 | 0.5138 |
| PI(20:3(5Z,8Z,11Z)/16:0) | 213.971 | 861.547 | 1.4558 | 0.00018 | 0.4158 |
| Monoethylhexyl phthalic acid | 6.154 | 279.159 | 1.1868 | 0.00227 | 0.5913 |
| PC(18:3(6Z,9Z,12Z)/18:0) | 159.042 | 784.585 | 1.3837 | 0.00054 | 0.5672 |
| Taurine | 307.770 | 126.022 | 1.3115 | 0.00426 | 0.5321 |
| Cer(d18:0/14:0) | 30.848 | 512.503 | 1.4224 | 0.00349 | 0.6241 |
| 3-[(5-Methyl-2-furanyl)methyl]-1H-pyrrole | 35.283 | 162.091 | 1.2829 | 0.00302 | 0.5448 |
| Tyrosyl-Gamma-glutamate | 309.401 | 310.129 | 1.3148 | 0.00137 | 0.3241 |
| 1-Deoxy-D-glucitol | 117.990 | 167.093 | 1.3258 | 0.00161 | 0.6200 |
| PC(P-18:1(9Z)/16:1(9Z)) | 156.921 | 742.573 | 1.3085 | 0.00096 | 0.4724 |
| PC(P-16:0/16:1(9Z)) | 162.488 | 716.560 | 1.1804 | 0.00648 | 0.4684 |
| PC(P-18:0/22:4(7Z,10Z,13Z,16Z)) | 152.237 | 822.637 | 1.1168 | 0.01709 | 0.6858 |
| Dihydrothymine | 440.888 | 129.066 | 1.3084 | 0.00069 | 0.6527 |
| PC(16:0/14:0) | 166.830 | 706.537 | 1.4645 | 0.00004 | 0.5362 |
| Oxonantenine | 399.584 | 336.087 | 1.3108 | 0.00150 | 0.6590 |
| Lysyl-Alanine | 446.724 | 218.150 | 1.1135 | 0.00883 | 0.6105 |
| Galactosylhydroxylysine | 540.273 | 325.160 | 1.3279 | 0.00102 | 0.6659 |
| PC(20:4(8Z,11Z,14Z,17Z)/20:3(5Z,8Z,11Z)) | 151.372 | 832.586 | 1.4520 | 0.00041 | 0.4579 |
| Tyrosyl-Proline | 290.427 | 279.134 | 1.3007 | 0.00329 | 0.5773 |
| Semilepidinoside B | 372.123 | 367.150 | 1.0287 | 0.04886 | 0.4244 |
| PC(22:6(4Z,7Z,10Z,13Z,16Z,19Z)/20:1(11Z)) | 149.595 | 860.616 | 1.2827 | 0.00263 | 0.6270 |
| PC(22:5(4Z,7Z,10Z,13Z,16Z)/16:0) | 153.931 | 808.586 | 1.4704 | 0.00006 | 0.5085 |
| PC(20:1(11Z)/15:0) | 161.656 | 774.597 | 1.1652 | 0.01290 | 0.5473 |
| PC(16:0/P-16:0) | 163.390 | 718.573 | 1.0516 | 0.01338 | 0.7573 |
| Thiomorpholine 3-carboxylate | 339.763 | 148.042 | 1.2083 | 0.00712 | 0.6769 |
| PC(14:0/14:0) | 169.409 | 678.506 | 1.4516 | 0.00011 | 0.4741 |
| Threoninyl-Leucine | 261.286 | 233.150 | 1.1939 | 0.03031 | 0.3816 |
| PC(20:4(8Z,11Z,14Z,17Z)/20:4(8Z,11Z,14Z,17Z)) | 183.274 | 830.554 | 1.4448 | 0.00000 | 0.1807 |
| SM(d18:1/18:1(9Z)) | 200.046 | 729.589 | 1.4134 | 0.00023 | 0.3299 |
| PC(22:6(4Z,7Z,10Z,13Z,16Z,19Z)/22:4(7Z,10Z,13Z,16Z)) | 148.293 | 882.600 | 1.2985 | 0.03119 | 0.1215 |
| PC(22:4(7Z,10Z,13Z,16Z)/14:0) | 60.430 | 782.571 | 1.1906 | 0.03152 | 0.4112 |
| Aspartyl-Aspartate | 475.413 | 249.072 | 1.0864 | 0.01100 | 0.6652 |
| PC(20:3(8Z,11Z,14Z)/20:0) | 38.774 | 840.646 | 1.0759 | 0.01318 | 0.7459 |
| Cer(d18:0/16:0) | 31.780 | 540.536 | 1.0786 | 0.03952 | 0.5358 |
| 8-Hydroxypinoresinol 4-glucoside | 399.523 | 537.195 | 1.1615 | 0.00870 | 0.7687 |
| PC(20:5(5Z,8Z,11Z,14Z,17Z)/20:3(5Z,8Z,11Z)) | 153.014 | 830.567 | 1.4185 | 0.00840 | 0.3123 |
| PC(22:5(4Z,7Z,10Z,13Z,16Z)/22:1(13Z)) | 148.728 | 890.662 | 1.3297 | 0.00082 | 0.5706 |
| PI(22:5(4Z,7Z,10Z,13Z,16Z)/16:0) | 210.612 | 885.548 | 1.4247 | 0.00033 | 0.5258 |
| Benzyl methyl sulfide | 32.737 | 128.107 | 1.3329 | 0.00027 | 0.3929 |
| SM(d18:1/12:0) | 205.418 | 647.511 | 1.3202 | 0.00162 | 0.5205 |
| L-Cyclo(alanylglycyl) | 373.883 | 129.066 | 1.4731 | 0.00006 | 0.4822 |
| PC(P-18:1(11Z)/20:3(5Z,8Z,11Z)) | 153.892 | 794.604 | 1.2190 | 0.00448 | 0.6832 |
| Hydroxyprolyl-Lysine | 301.528 | 260.160 | 1.0727 | 0.03491 | 0.4367 |
| PC(22:6(4Z,7Z,10Z,13Z,16Z,19Z)/20:3(5Z,8Z,11Z)) | 37.914 | 856.583 | 1.4431 | 0.00339 | 0.1866 |
| 3-Aminopropionaldehyde | 56.979 | 74.060 | 1.4420 | 0.00007 | 0.6366 |
| PE(P-18:1(11Z)/22:5(4Z,7Z,10Z,13Z,16Z)) | 150.012 | 776.557 | 1.1626 | 0.00903 | 0.6903 |
| PE(22:6(4Z,7Z,10Z,13Z,16Z,19Z)/P-16:0) | 152.130 | 748.528 | 1.1064 | 0.01631 | 0.7353 |
| PC(22:6(4Z,7Z,10Z,13Z,16Z,19Z)/20:0) | 151.319 | 862.633 | 1.0406 | 0.02709 | 0.7478 |
| PC(18:1(11Z)/14:0) | 76.904 | 732.555 | 1.4527 | 0.00016 | 0.4468 |
| PC(P-18:1(11Z)/22:5(4Z,7Z,10Z,13Z,16Z)) | 168.544 | 818.591 | 1.5121 | 0.00000 | 0.4393 |
| PE(18:3(9Z,12Z,15Z)/16:0) | 167.680 | 714.505 | 1.1845 | 0.00444 | 0.6760 |
| PC(16:1(9Z)/14:0) | 67.444 | 704.521 | 1.3575 | 0.00429 | 0.3558 |
| Phenylbutyrylglutamine | 222.828 | 293.150 | 1.1218 | 0.00717 | 0.6535 |
| L-Hexanoylcarnitine | 243.631 | 260.186 | 1.4766 | 0.00733 | 0.1110 |
| PC(22:5(4Z,7Z,10Z,13Z,16Z)/20:5(5Z,8Z,11Z,14Z,17Z)) | 148.724 | 854.568 | 1.3962 | 0.01254 | 0.2278 |
| SM(d18:1/14:0) | 203.521 | 675.545 | 1.5194 | 0.00000 | 0.5808 |
| PC(16:0/14:1(9Z)) | 166.784 | 704.521 | 1.4687 | 0.00171 | 0.4049 |
| PC(20:4(8Z,11Z,14Z,17Z)/P-18:0) | 38.775 | 794.606 | 1.2687 | 0.00082 | 0.6197 |
| 3-Methyluridine | 469.195 | 259.092 | 1.1576 | 0.00587 | 0.6058 |
| stearoyl sphingomyelin | 200.046 | 731.605 | 1.3352 | 0.00131 | 0.4102 |
| Asparaginyl-Proline | 381.972 | 230.114 | 1.4199 | 0.00000 | 0.4075 |
| 1-(1-Pyrrolidinyl)-2-butanone | 32.731 | 142.122 | 1.2569 | 0.01021 | 0.5554 |
| PC(24:1(15Z)/18:4(6Z,9Z,12Z,15Z)) | 148.734 | 864.648 | 1.0477 | 0.02662 | 0.6846 |
| PC(22:5(4Z,7Z,10Z,13Z,16Z)/22:6(4Z,7Z,10Z,13Z,16Z,19Z)) | 147.811 | 880.585 | 1.4252 | 0.01883 | 0.1557 |
| PC(22:6(4Z,7Z,10Z,13Z,16Z,19Z)/20:4(5Z,8Z,11Z,14Z)) | 37.919 | 854.568 | 1.3328 | 0.00688 | 0.1861 |
| 2-Azetidinecarboxylic acid | 410.086 | 102.055 | 1.4639 | 0.00001 | 0.4885 |
| PC(16:1(9Z)/16:1(9Z)) | 164.248 | 730.537 | 1.5231 | 0.00002 | 0.4565 |
| Norophthalmic acid | 349.304 | 276.119 | 1.1549 | 0.02012 | 0.7235 |
| Alkaloid RC | 488.940 | 532.187 | 1.2241 | 0.00648 | 0.5262 |
| N-Nitroso-pyrrolidine | 34.412 | 101.060 | 1.1057 | 0.02896 | 0.5474 |
| PC(22:5(7Z,10Z,13Z,16Z,19Z)/20:4(5Z,8Z,11Z,14Z)) | 149.693 | 856.584 | 1.4098 | 0.01160 | 0.2182 |
| lysoPC(26:0) | 170.285 | 636.497 | 1.3752 | 0.00036 | 0.4512 |
| N-Acetylglutamine | 410.056 | 189.087 | 1.4955 | 0.00000 | 0.4748 |
| Alanyl-Isoleucine | 411.835 | 203.139 | 1.4736 | 0.00001 | 0.5229 |
| Cer(d18:1/22:1(13Z)) | 31.862 | 620.597 | 1.2671 | 0.00283 | 0.7357 |
| PS(18:0/22:6(4Z,7Z,10Z,13Z,16Z,19Z)) | 212.229 | 836.542 | 1.3717 | 0.00049 | 0.6204 |
| L-Theanine | 56.125 | 175.107 | 1.1021 | 0.02227 | 0.6617 |
| 5-Hydroxy-4-methoxy-6-canthinone 3-N-oxide | 246.644 | 283.069 | 1.2986 | 0.00689 | 0.2035 |
| PC(20:3(8Z,11Z,14Z)/14:0) | 162.531 | 756.555 | 1.2230 | 0.00401 | 0.5169 |
| Santene | 31.872 | 123.117 | 1.3289 | 0.00986 | 0.3881 |
| PC(18:3(6Z,9Z,12Z)/15:0) | 201.779 | 742.537 | 1.2809 | 0.00054 | 0.5218 |
| PE(P-18:1(11Z)/22:4(7Z,10Z,13Z,16Z)) | 151.309 | 778.573 | 1.0181 | 0.04712 | 0.6922 |
| PS(22:4(7Z,10Z,13Z,16Z)/18:1(9Z)) | 212.325 | 838.559 | 1.1624 | 0.01264 | 0.6545 |
| Ethyl 3-mercaptopropanoic acid | 416.547 | 135.048 | 1.4416 | 0.00028 | 0.3990 |
| (Â±)-Glycerol 1,2-diacetate | 361.625 | 177.074 | 1.4340 | 0.00020 | 0.2529 |
| Palmitic acid | 40.499 | 274.273 | 1.1245 | 0.01473 | 0.7392 |
| 26-Methyl nigranoate | 43.966 | 485.358 | 1.4261 | 0.00007 | 0.6088 |
