# Supplementary material for: Multi-Omics Analysis of Mammary Metabolic Changes in Dairy Cows Exposed to Hypoxia
Source: Front Vet Sci. 2021 Oct 14;8:764135. doi: 10.3389/fvets.2021.764135 (PMC8553012; doi:10.3389/fvets.2021.764135)
Supplement: Supplementary file 2 [file Data_Sheet_3.docx]

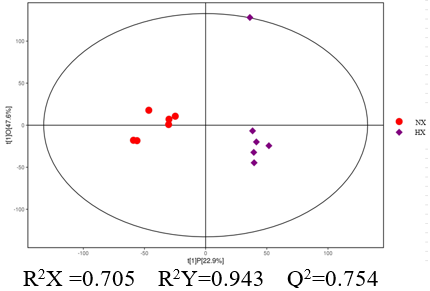


**Fig. S1**. PLS-DA score plot of the two groups of BMECs using the identified metabolites (lipidomics) in negative ionization mode. “NX and HX” represent control, hypoxia group, respectively.

**
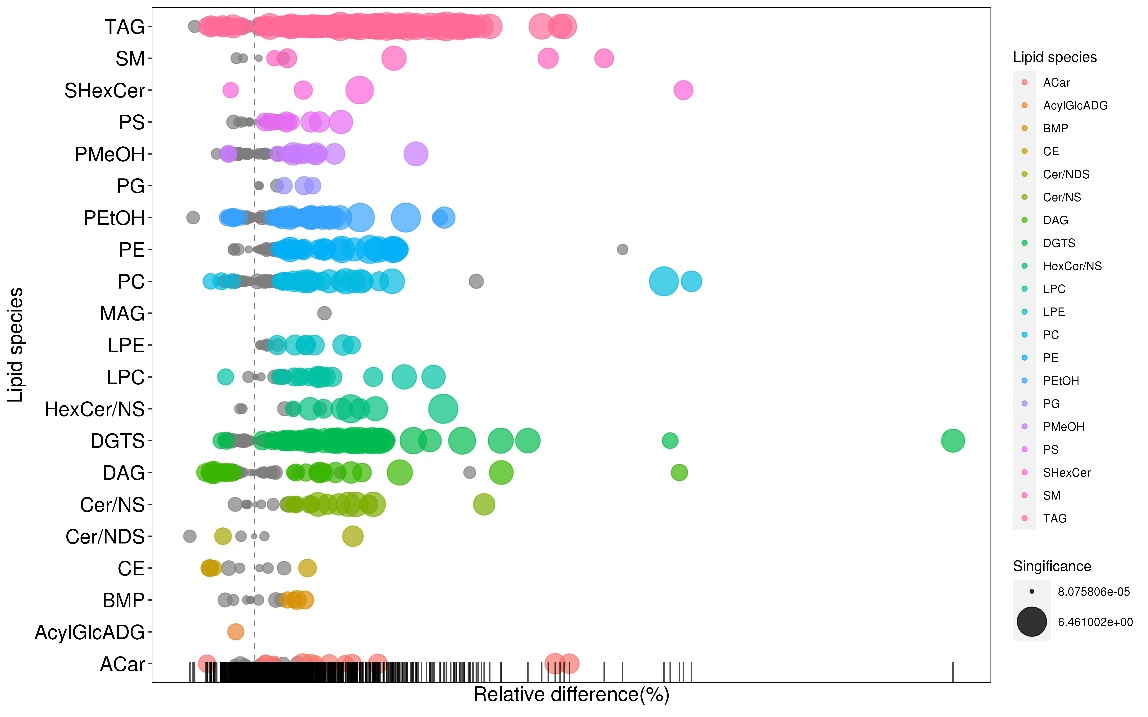
**

**Fig. S2**. Bubble plot for the two groups of BMECs using the identified metabolites (lipidomics) in positive ionization mode.
