# Supplementary figures and images for: Multi-Omics Analysis of Mammary Metabolic Changes in Dairy Cows Exposed to Hypoxia
Source: Front Vet Sci. 2021 Oct 14;8:764135. doi: 10.3389/fvets.2021.764135 (PMC8553012; doi:10.3389/fvets.2021.764135)

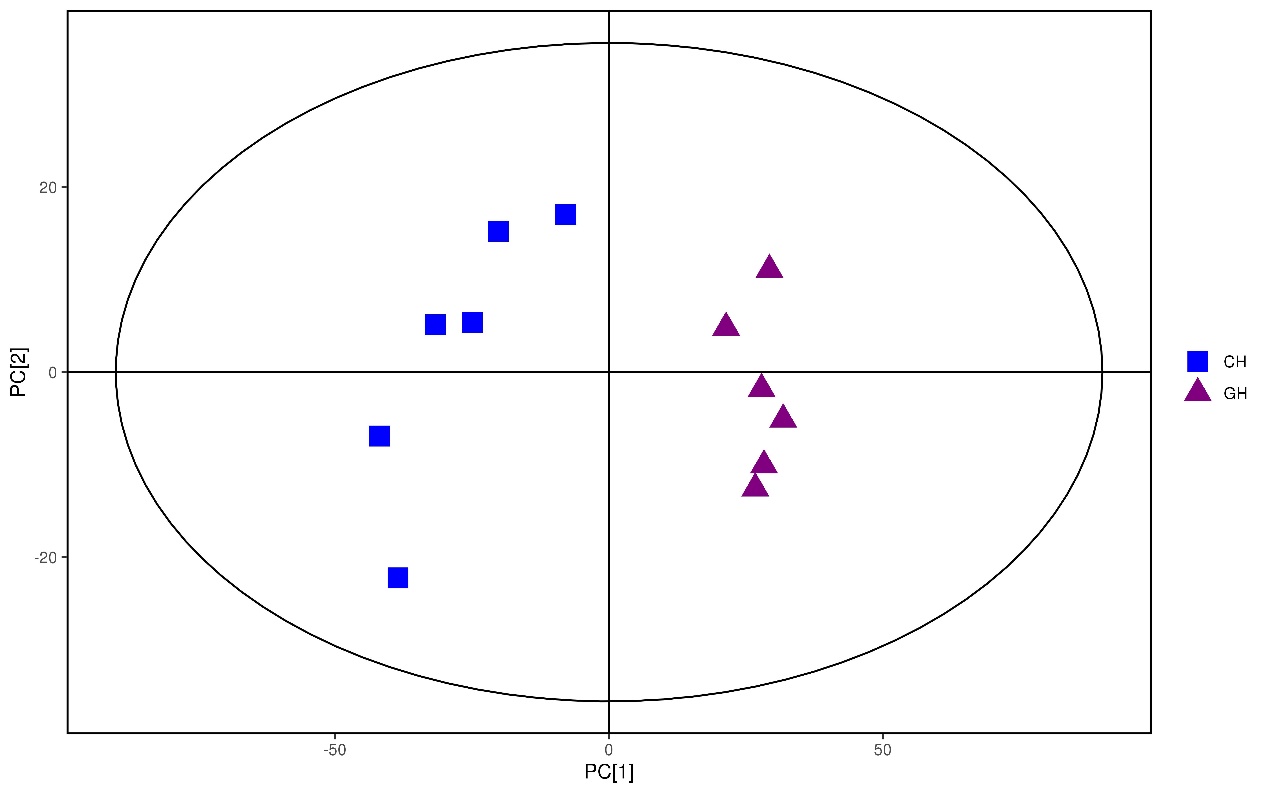


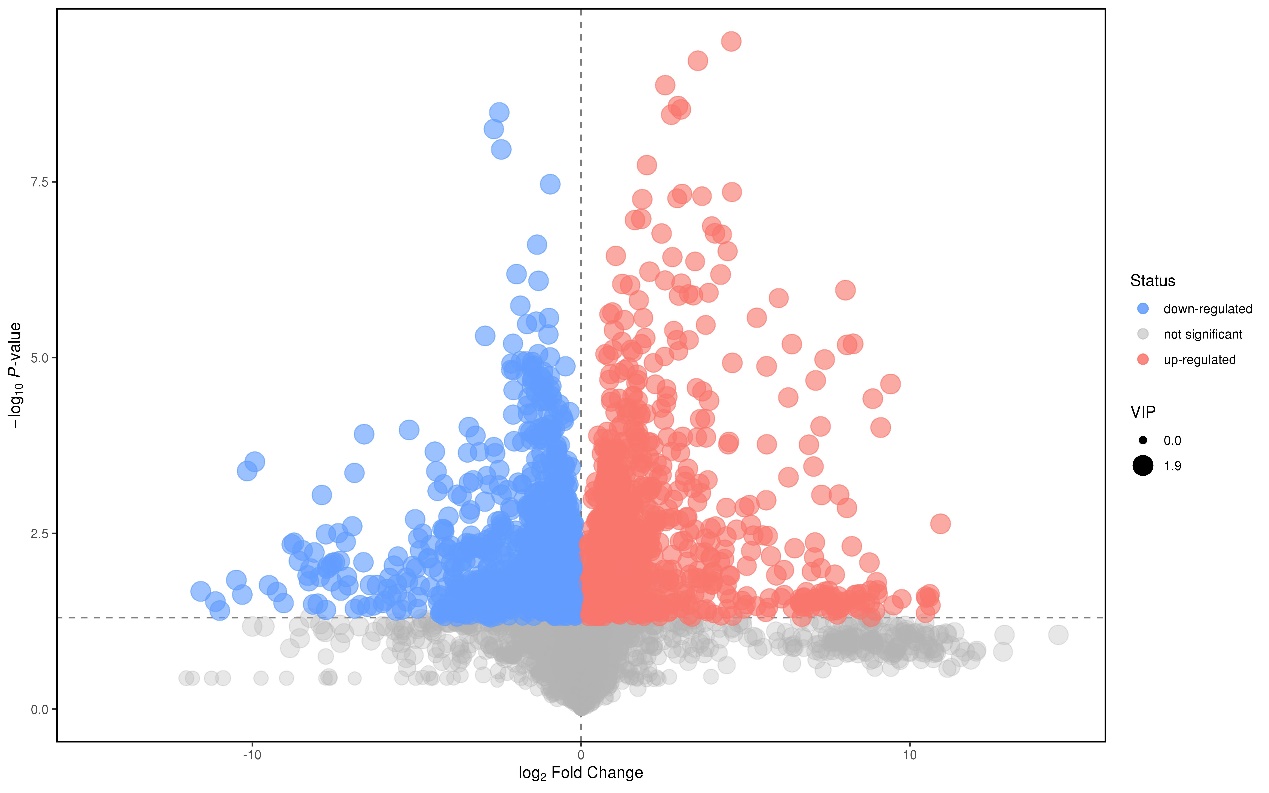


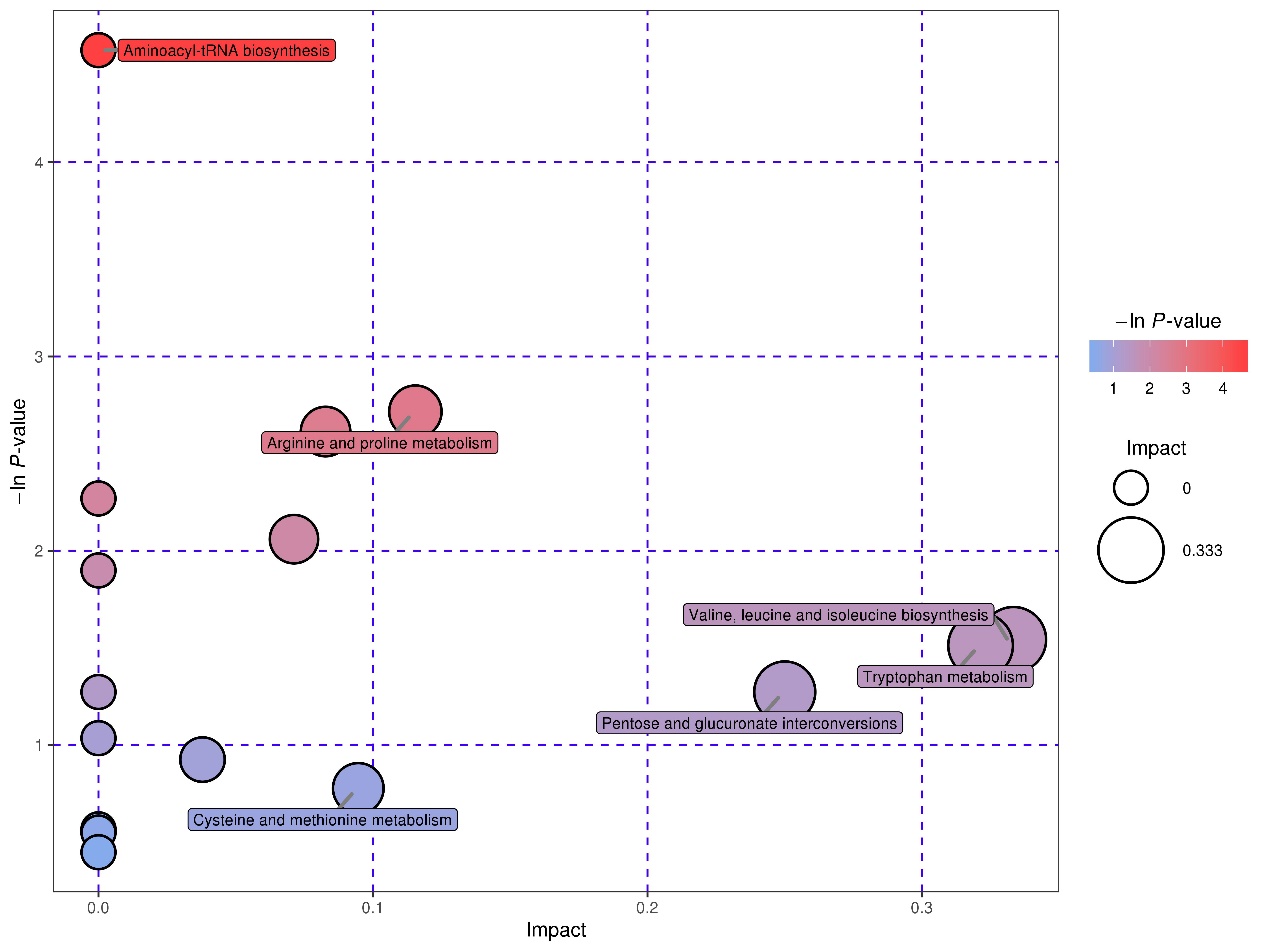

Supplement: Supplementary file 3 [file Data_Sheet_1.ZIP › metabonomics for blood.docx]

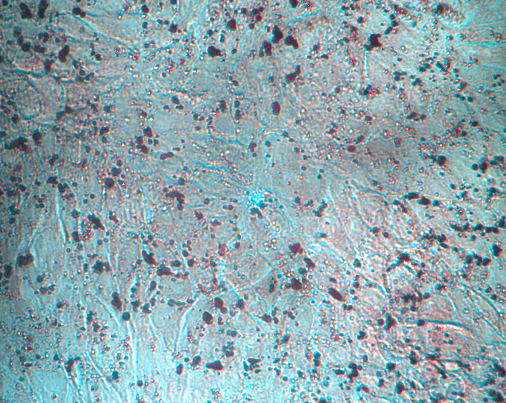

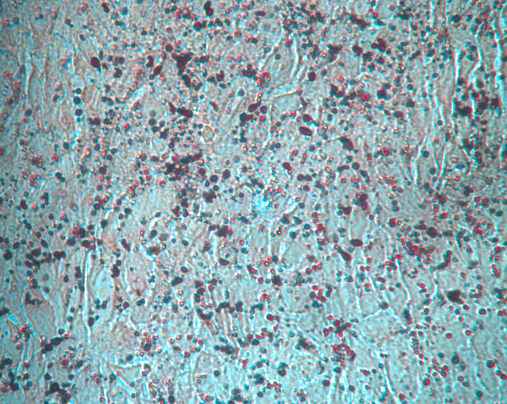


NX (400X) HX (400X)


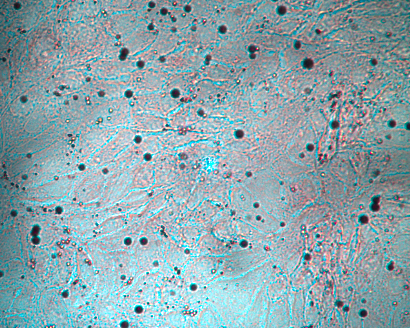

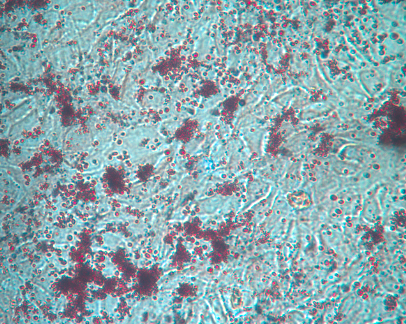

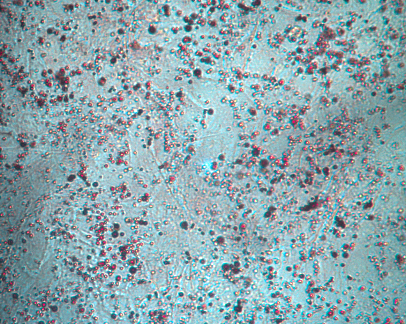


NX (400X) HX (400X) HX +shAGPAT2 (400X)

Supplement: Supplementary file 4 [file Data_Sheet_2.ZIP › raw data for cells/raw data/ORO.docx]

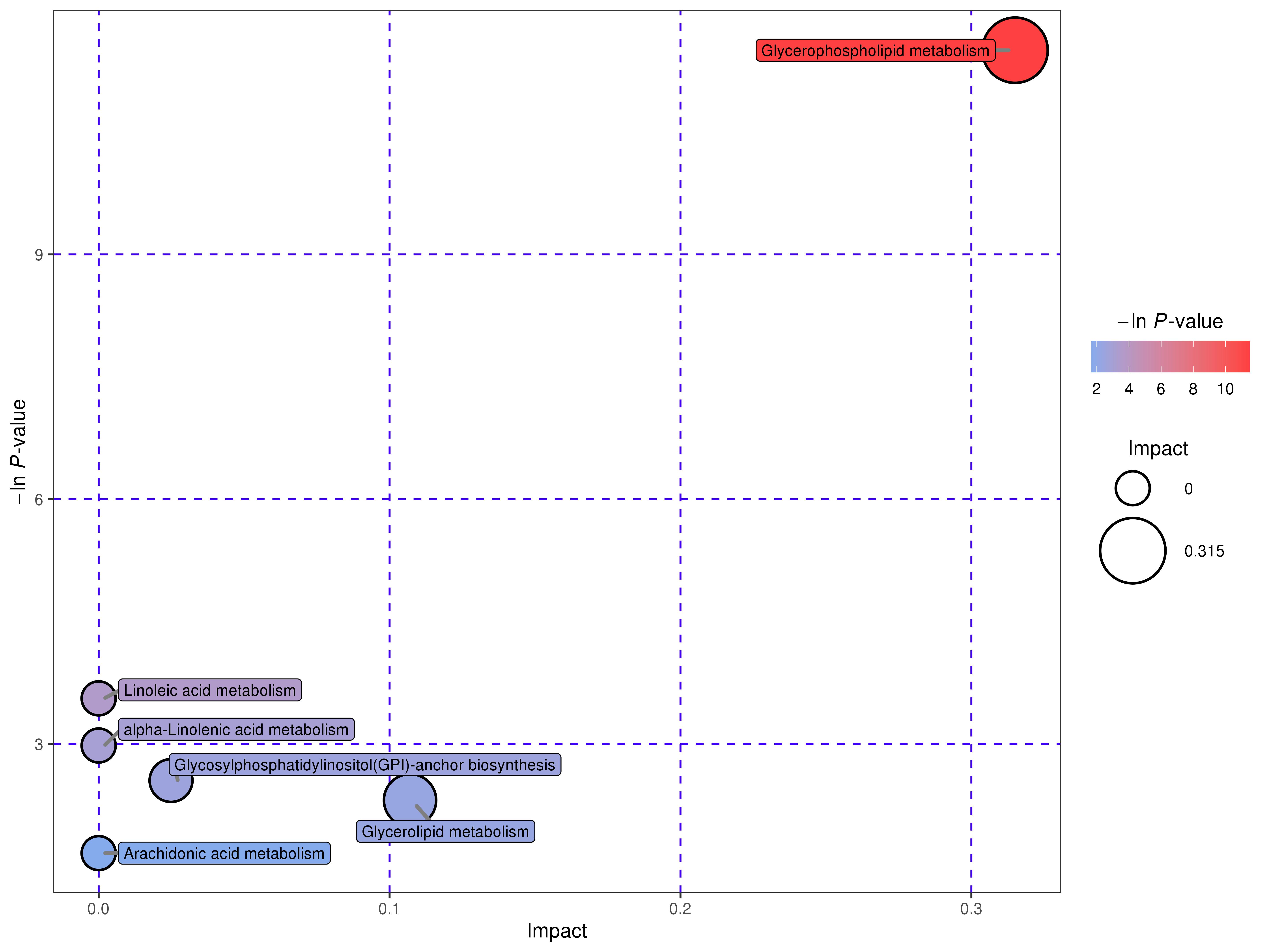

Supplement: Supplementary file 4 [file Data_Sheet_2.ZIP › raw data for cells/raw data/POS for lipidomics/POS-Pathway Analysis/Pathway Analysis/HX VS NX/Bubble Plot.jpg]

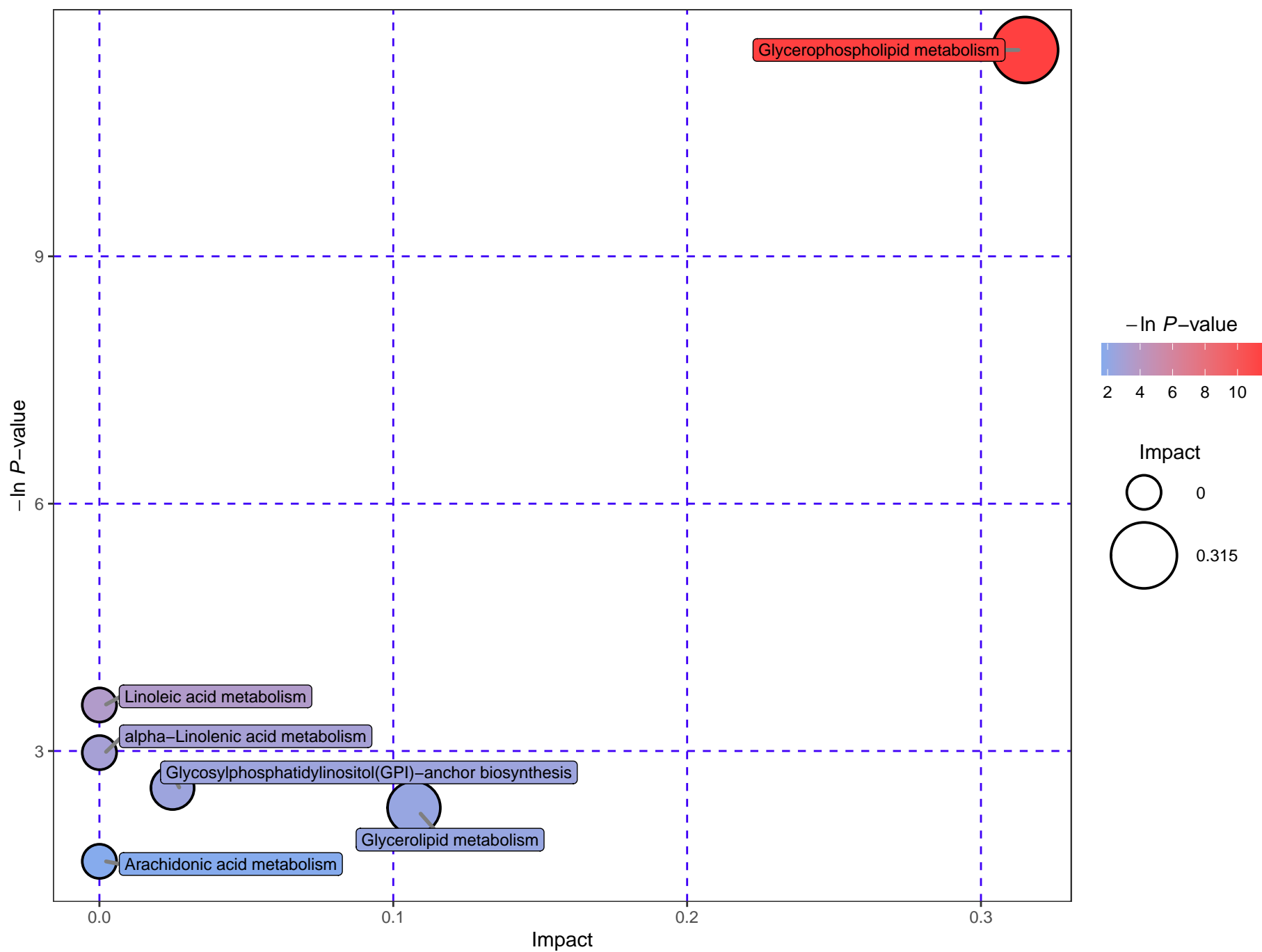

Supplement: Supplementary file 4 [file Data_Sheet_2.ZIP › raw data for cells/raw data/POS for lipidomics/POS-Pathway Analysis/Pathway Analysis/HX VS NX/Bubble Plot.pdf]

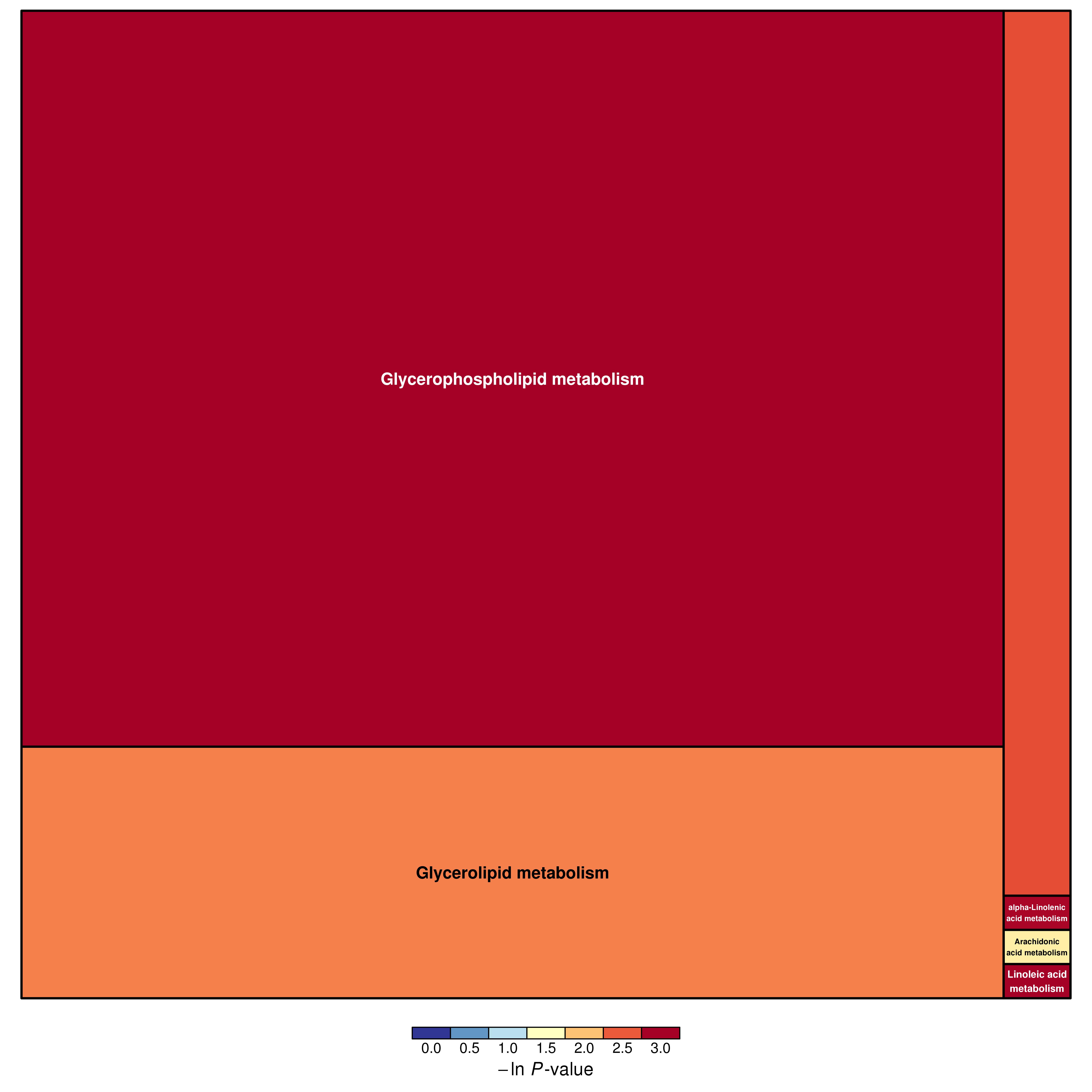

Supplement: Supplementary file 4 [file Data_Sheet_2.ZIP › raw data for cells/raw data/POS for lipidomics/POS-Pathway Analysis/Pathway Analysis/HX VS NX/treemap.jpg]

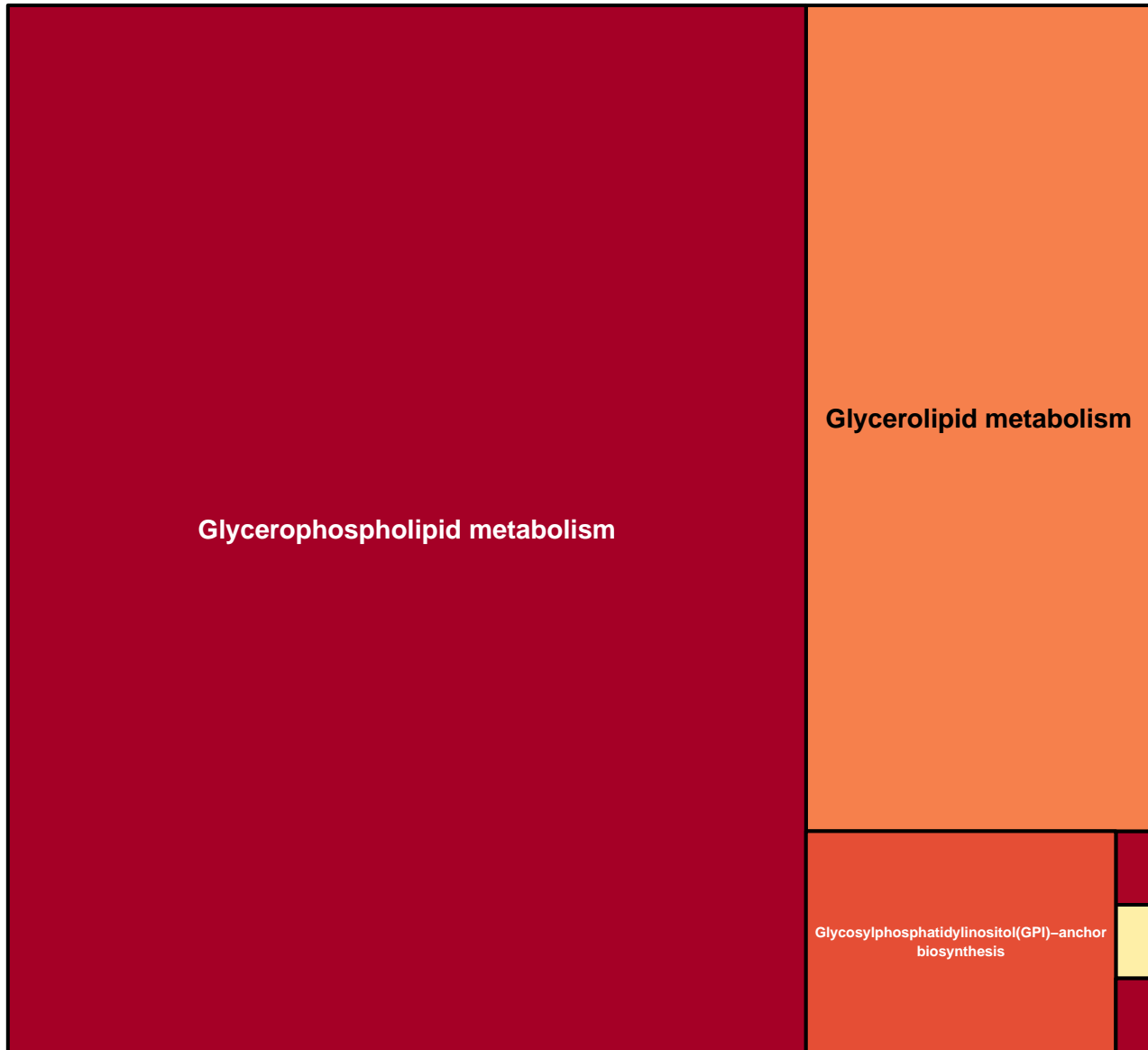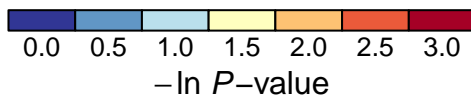

Supplement: Supplementary file 4 [file Data_Sheet_2.ZIP › raw data for cells/raw data/POS for lipidomics/POS-Pathway Analysis/Pathway Analysis/HX VS NX/treemap.pdf]

Intercepts:  $R^2Y(\text{cum}) = (0, 0.84)$ ,  $Q^2(\text{cum}) = (0, -0.8)$

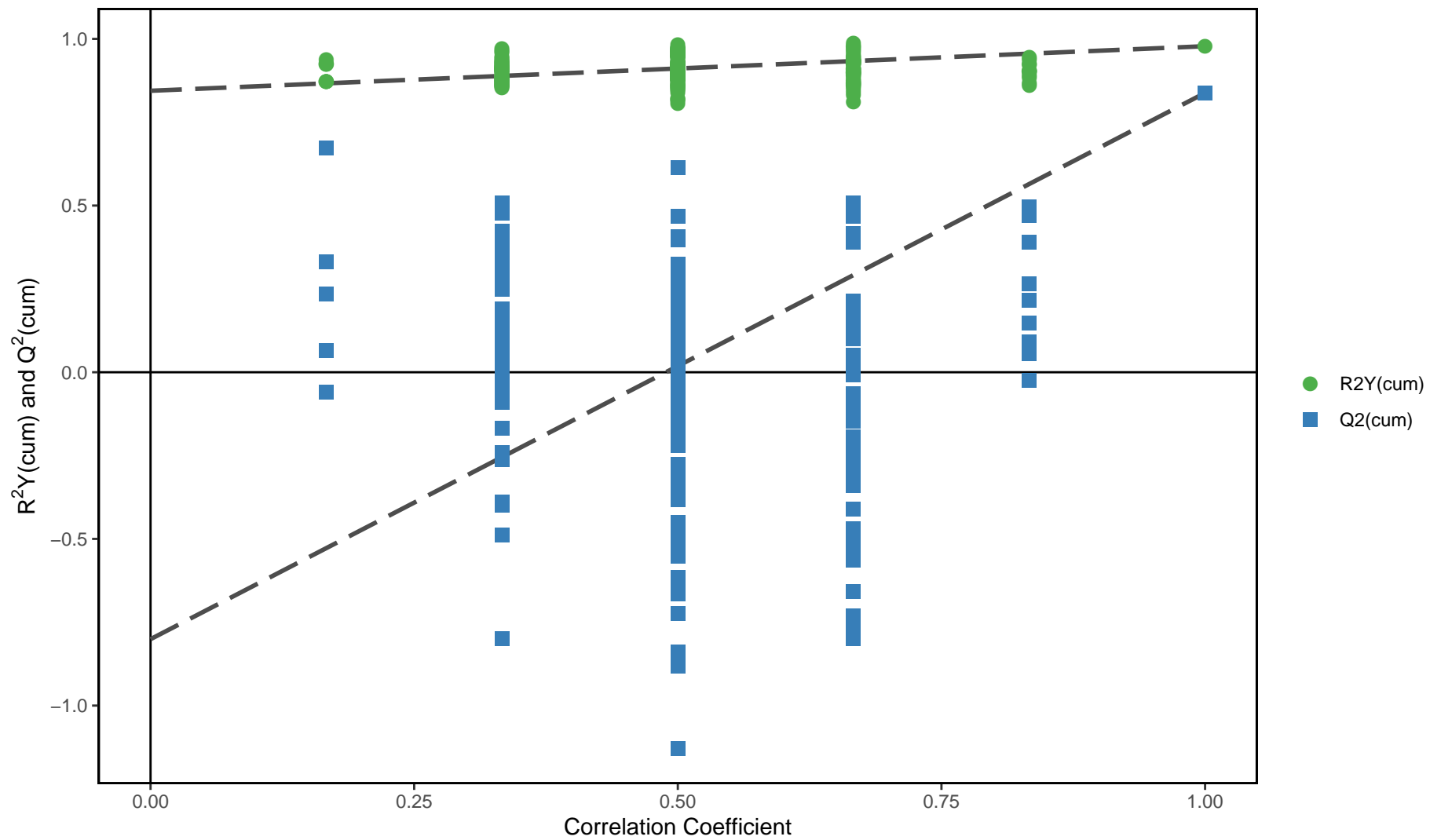

Supplement: Supplementary file 4 [file Data_Sheet_2.ZIP › raw data for cells/raw data/POS for lipidomics/Statistical Analysis/HX VS NX/OPLS-DA permutation plot.pdf]

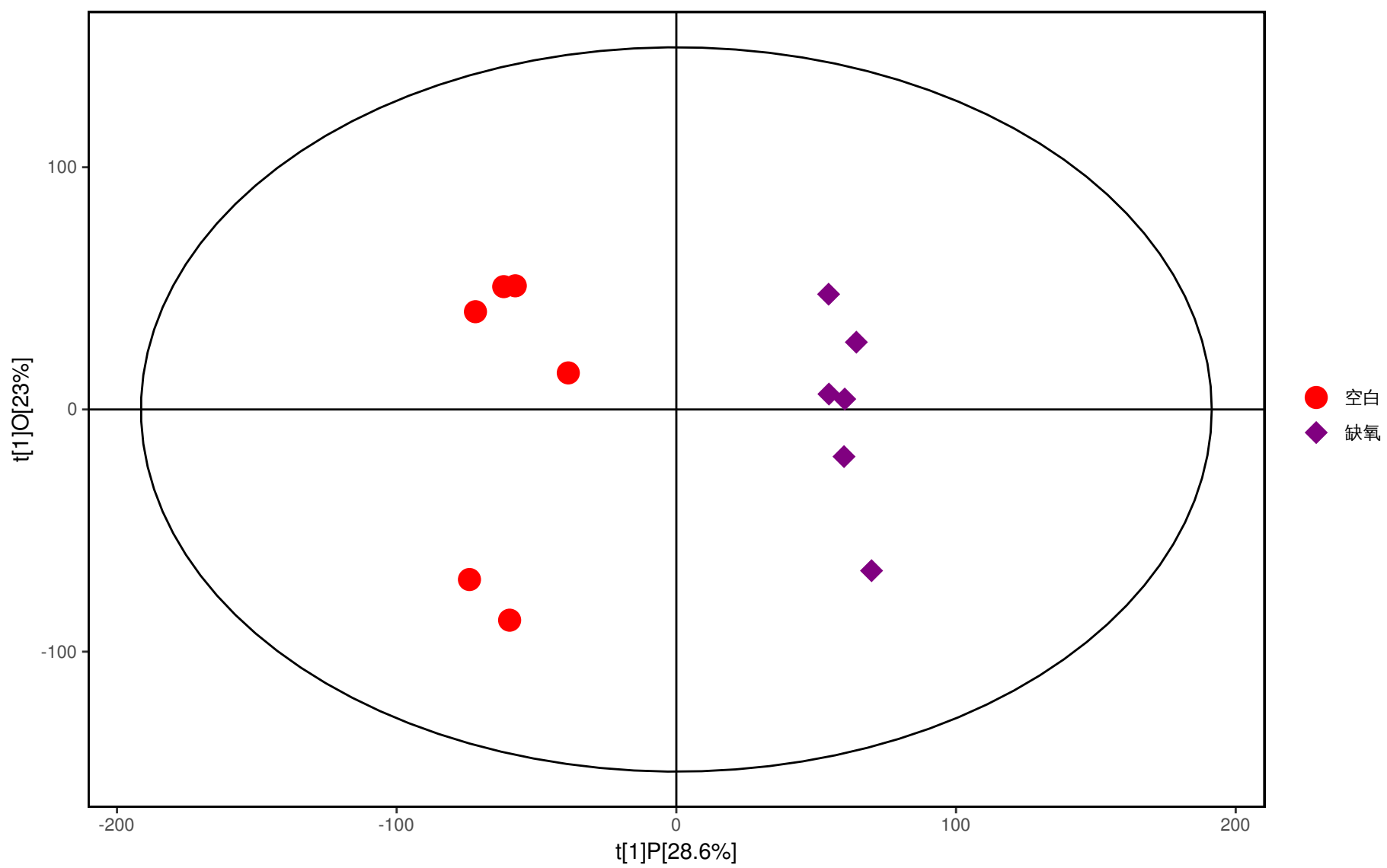

Supplement: Supplementary file 4 [file Data_Sheet_2.ZIP › raw data for cells/raw data/POS for lipidomics/Statistical Analysis/HX VS NX/OPLS-DA score plot.pdf]

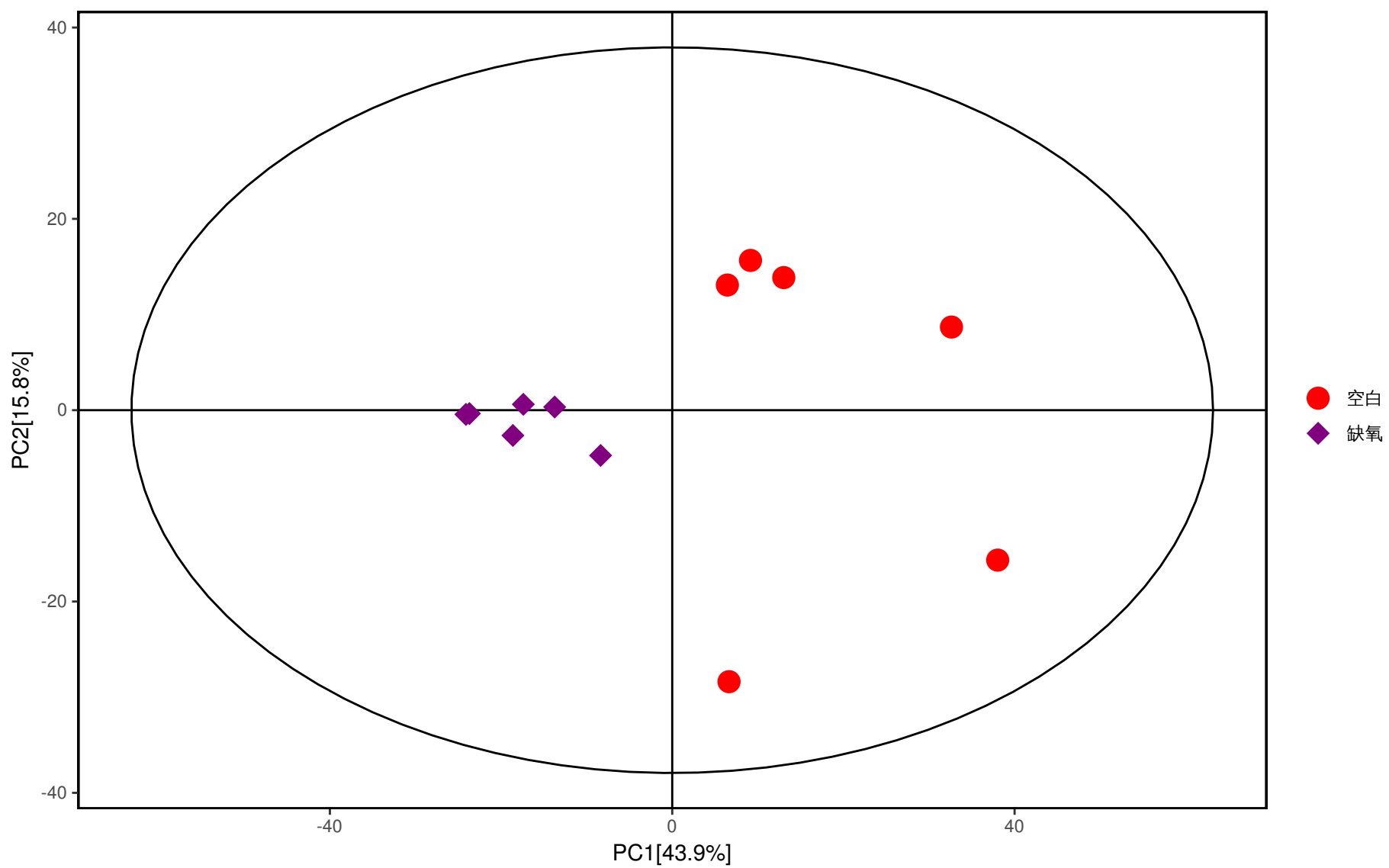

Supplement: Supplementary file 4 [file Data_Sheet_2.ZIP › raw data for cells/raw data/POS for lipidomics/Statistical Analysis/HX VS NX/PCA score plot.pdf]

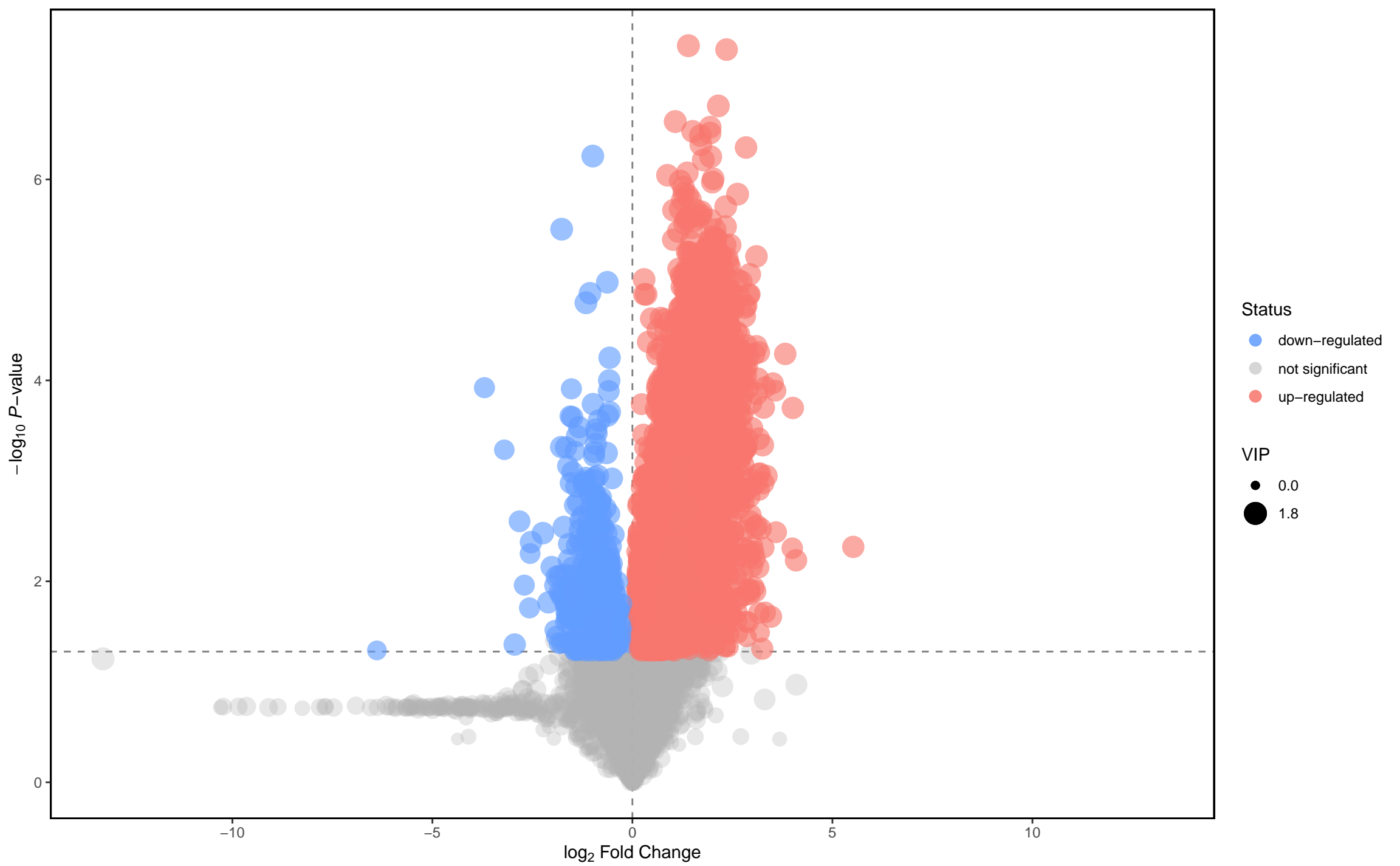

Supplement: Supplementary file 4 [file Data_Sheet_2.ZIP › raw data for cells/raw data/POS for lipidomics/Statistical Analysis/HX VS NX/volcano plot.pdf]

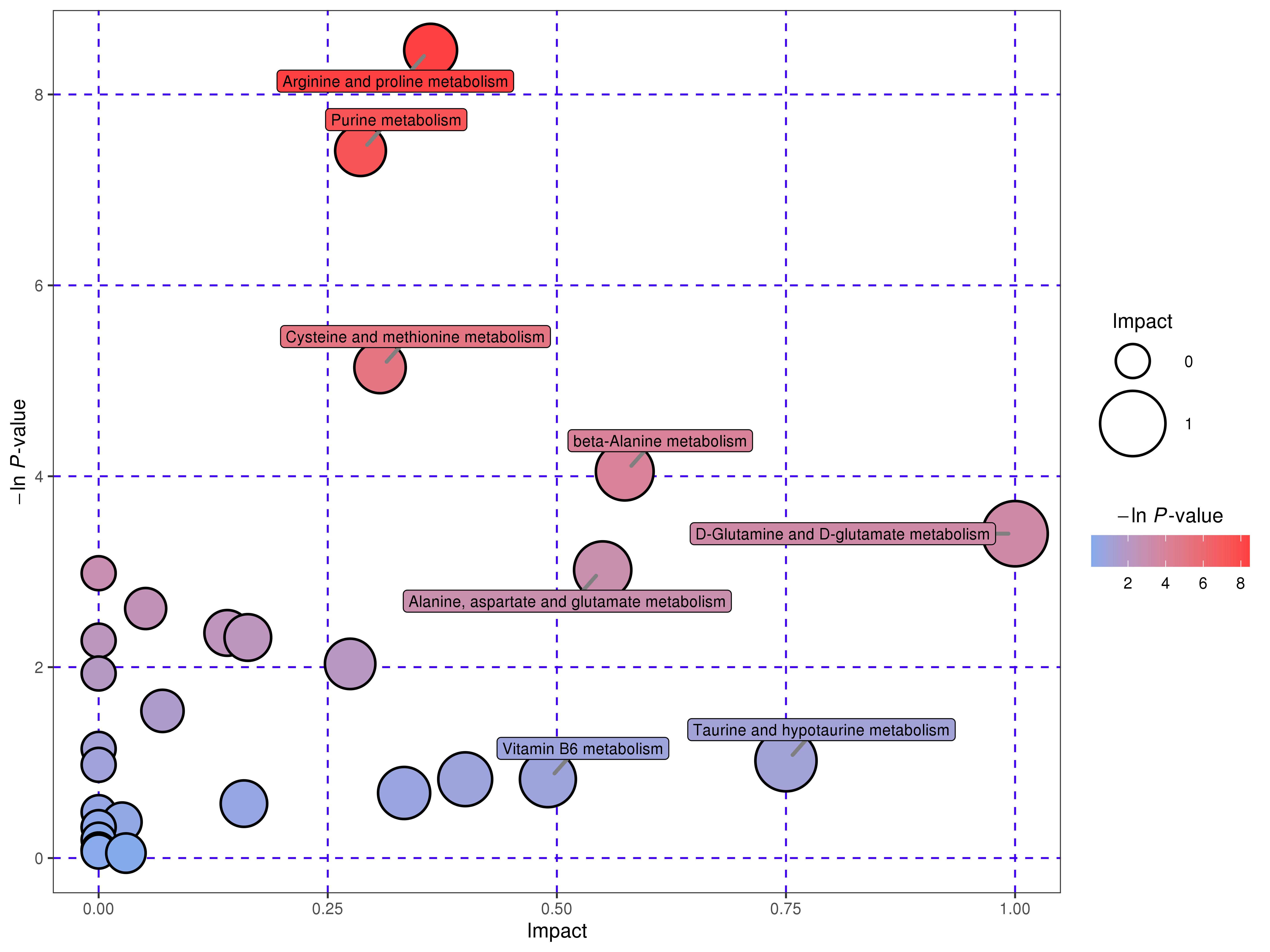

Supplement: Supplementary file 4 [file Data_Sheet_2.ZIP › raw data for cells/raw data/POS-for metabolomics/Pathway Analysis/HX VS NX/Bubble Plot.jpg]

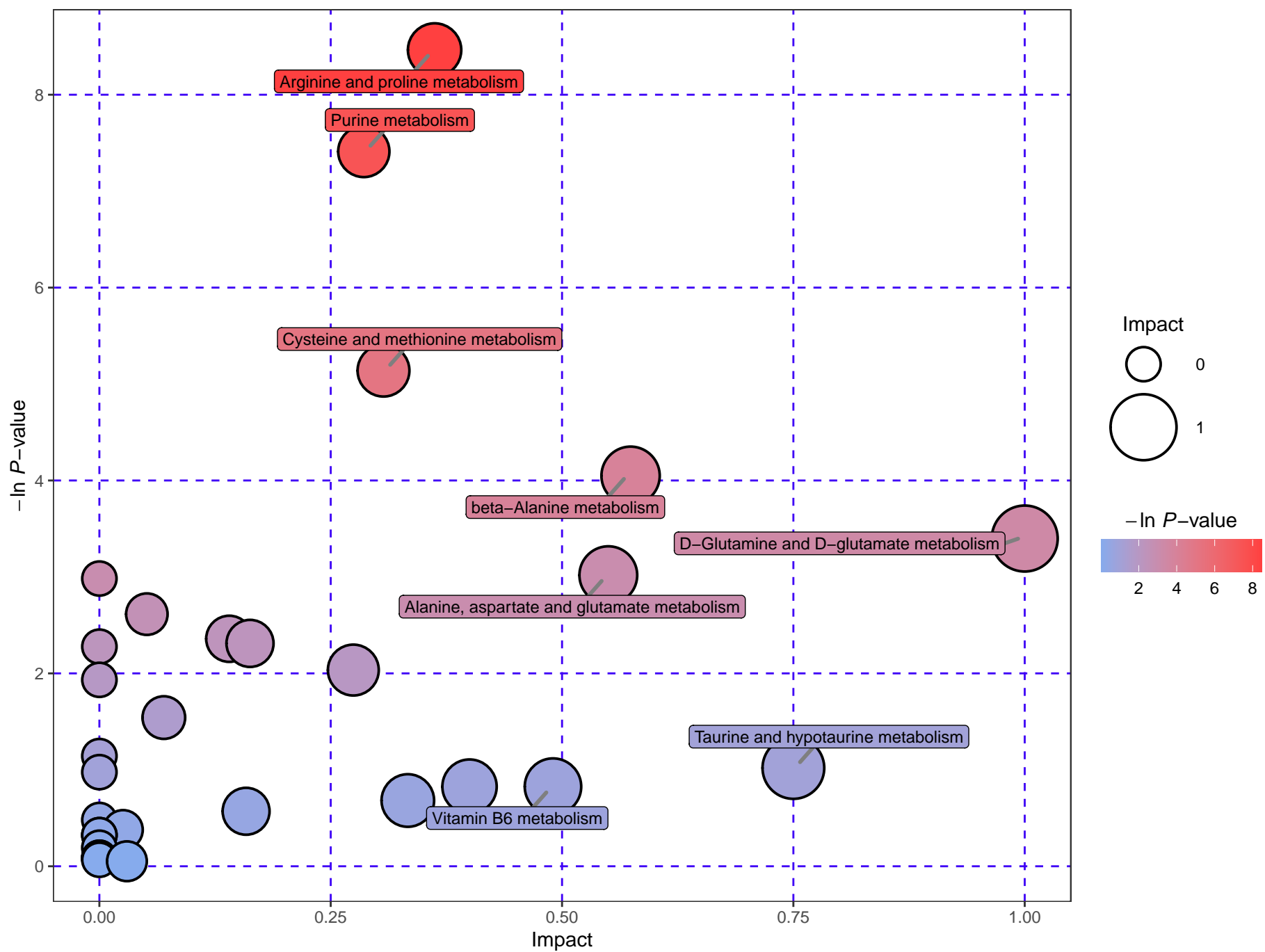

Supplement: Supplementary file 4 [file Data_Sheet_2.ZIP › raw data for cells/raw data/POS-for metabolomics/Pathway Analysis/HX VS NX/Bubble Plot.pdf]

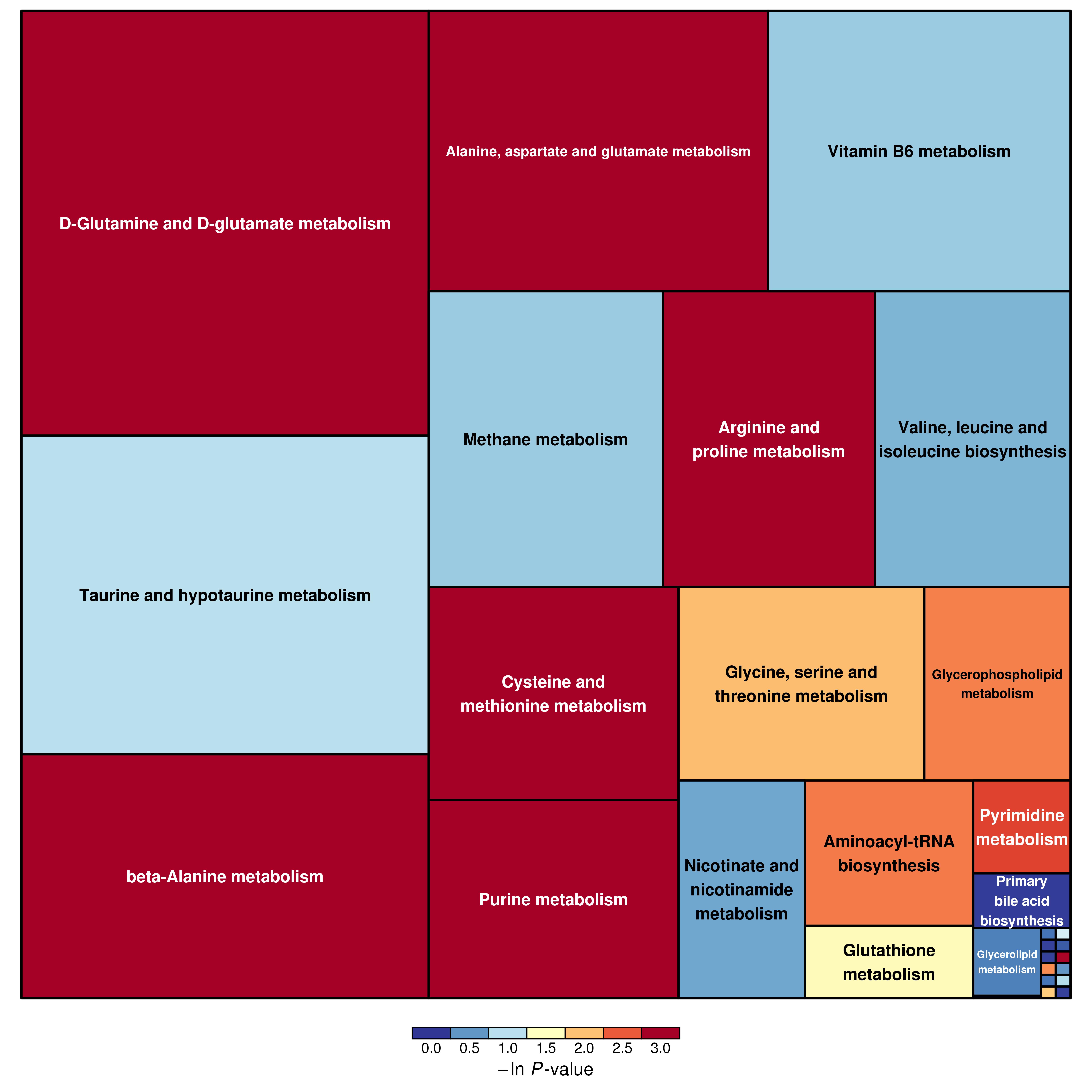

Supplement: Supplementary file 4 [file Data_Sheet_2.ZIP › raw data for cells/raw data/POS-for metabolomics/Pathway Analysis/HX VS NX/treemap.jpg]

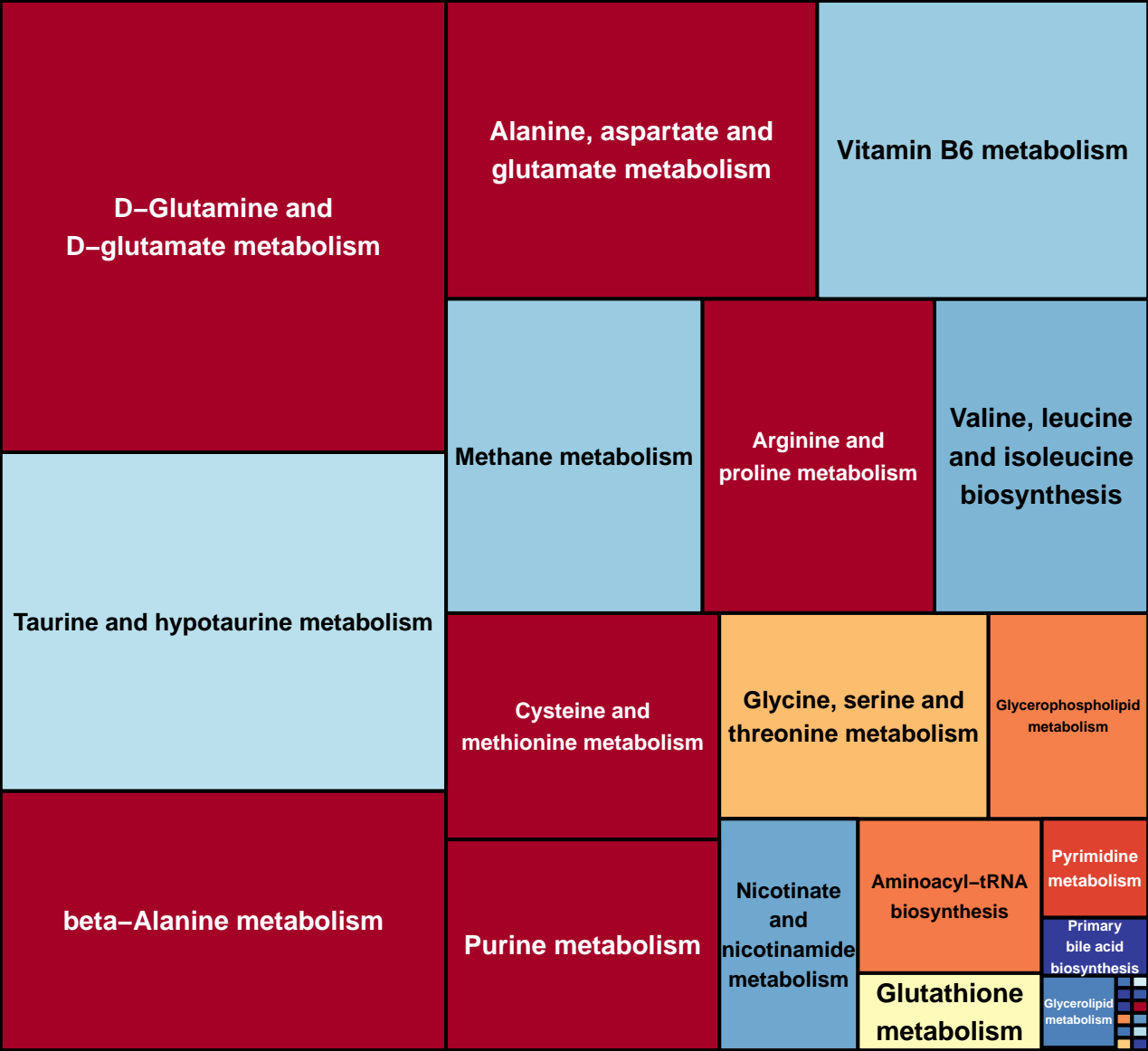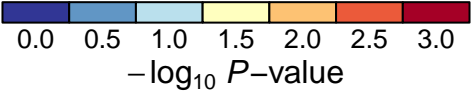

Supplement: Supplementary file 4 [file Data_Sheet_2.ZIP › raw data for cells/raw data/POS-for metabolomics/Pathway Analysis/HX VS NX/treemap.pdf]

Intercepts:  $R^2Y(\text{cum}) = (0, 0.86)$ ,  $Q^2(\text{cum}) = (0, -0.87)$

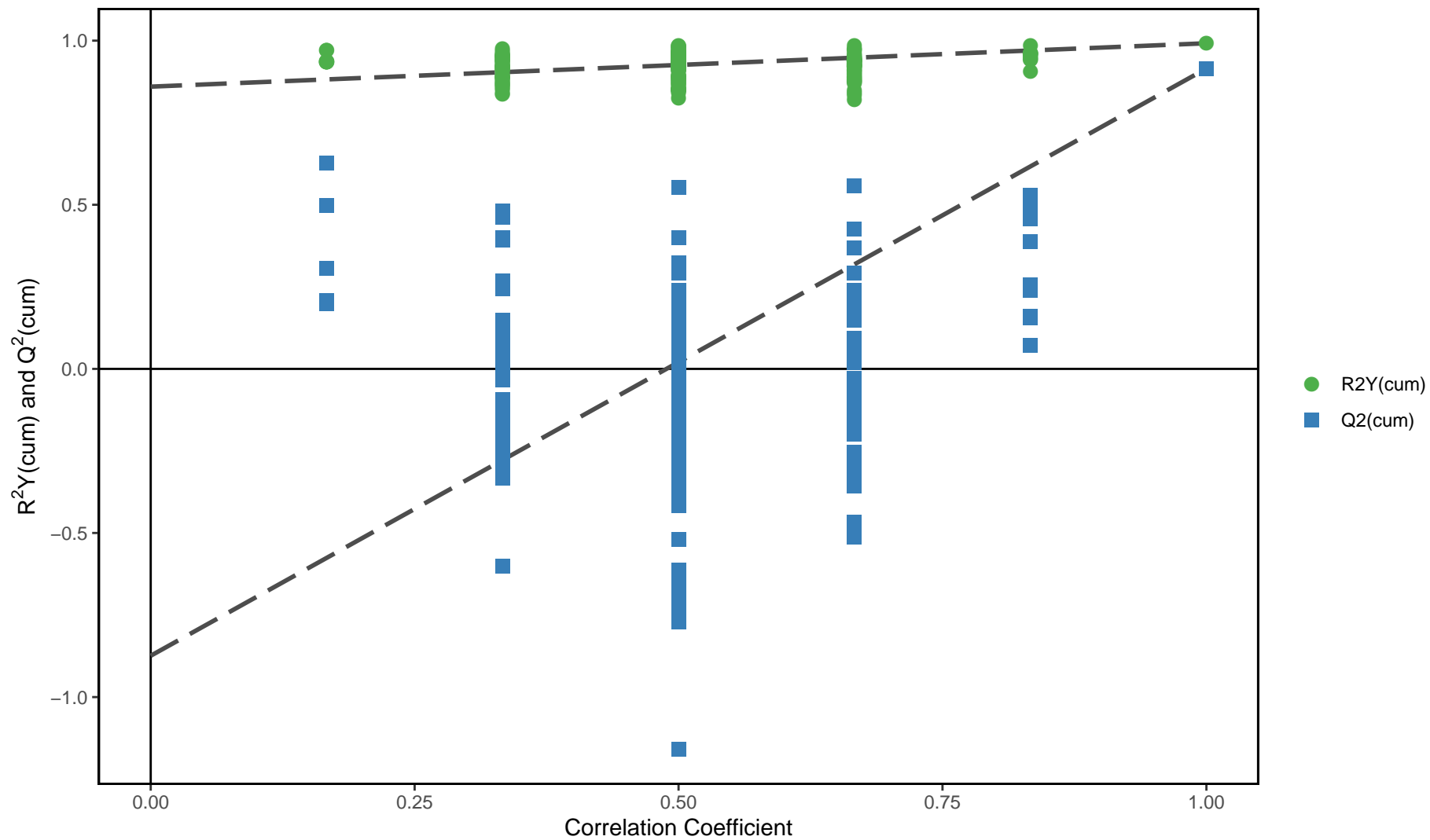

Supplement: Supplementary file 4 [file Data_Sheet_2.ZIP › raw data for cells/raw data/POS-for metabolomics/Statistical Analysis/HX VS NX/OPLS-DA permutation plot.pdf]

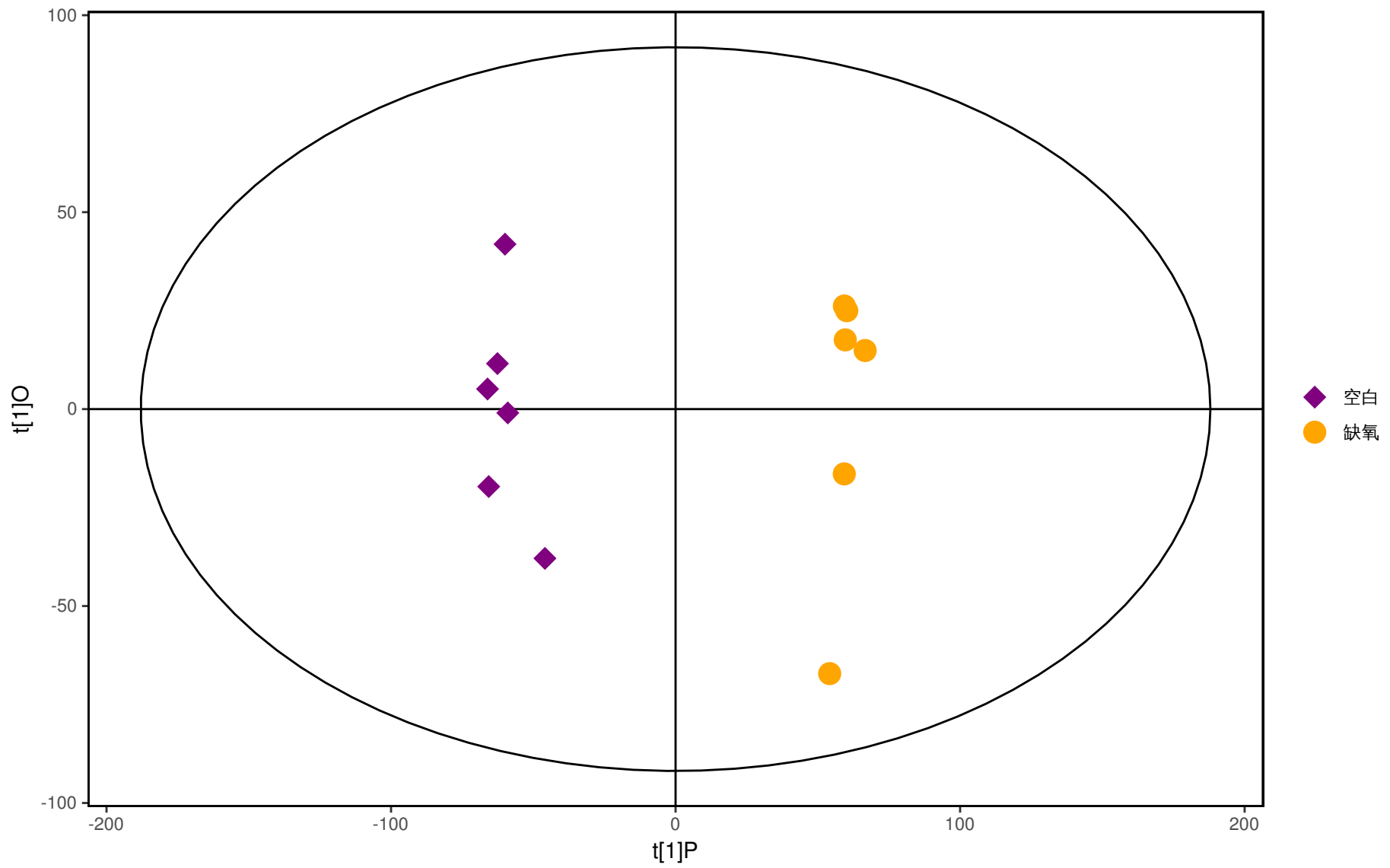

Supplement: Supplementary file 4 [file Data_Sheet_2.ZIP › raw data for cells/raw data/POS-for metabolomics/Statistical Analysis/HX VS NX/OPLS-DA score plot.pdf]

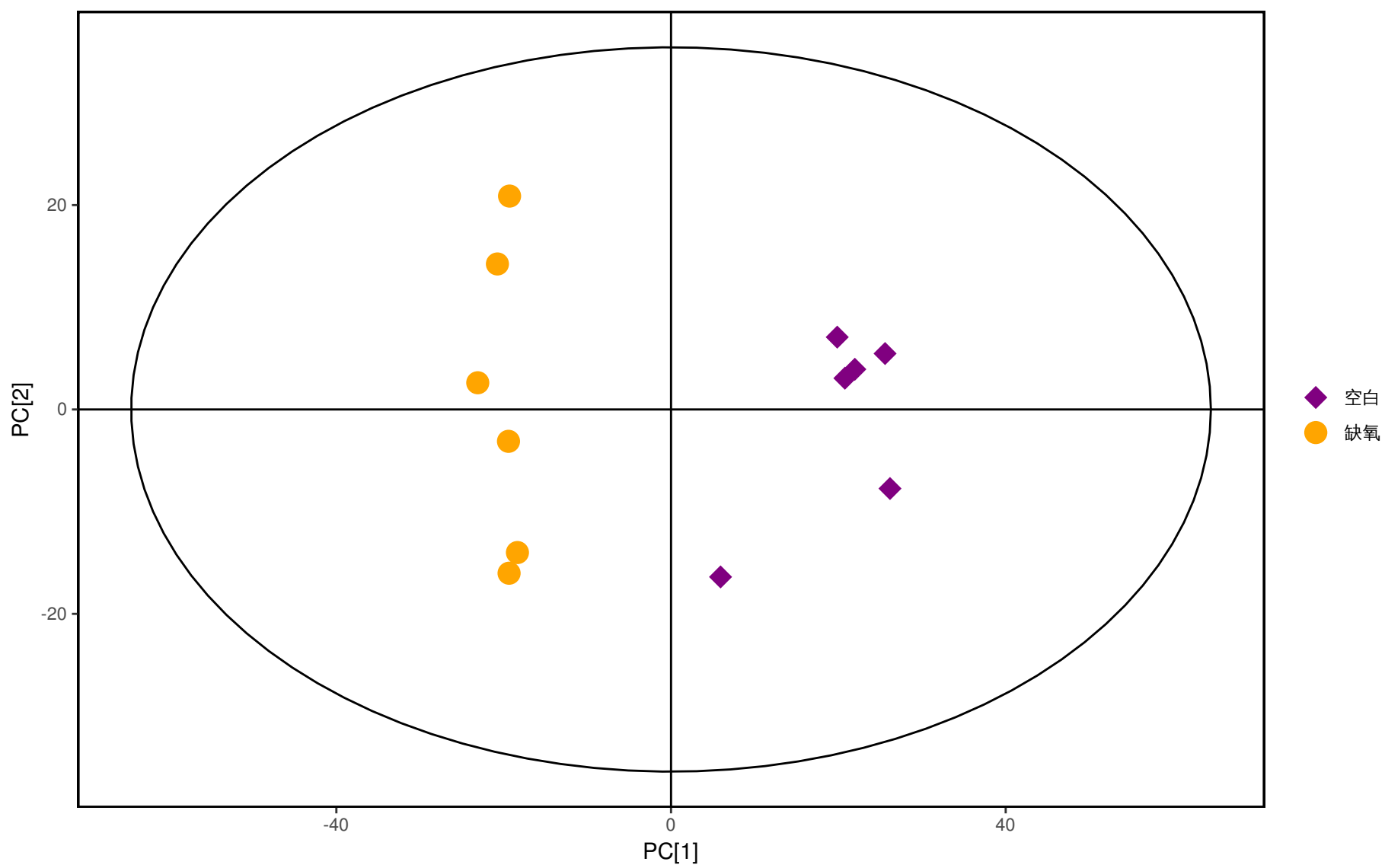

Supplement: Supplementary file 4 [file Data_Sheet_2.ZIP › raw data for cells/raw data/POS-for metabolomics/Statistical Analysis/HX VS NX/PCA score plot.pdf]

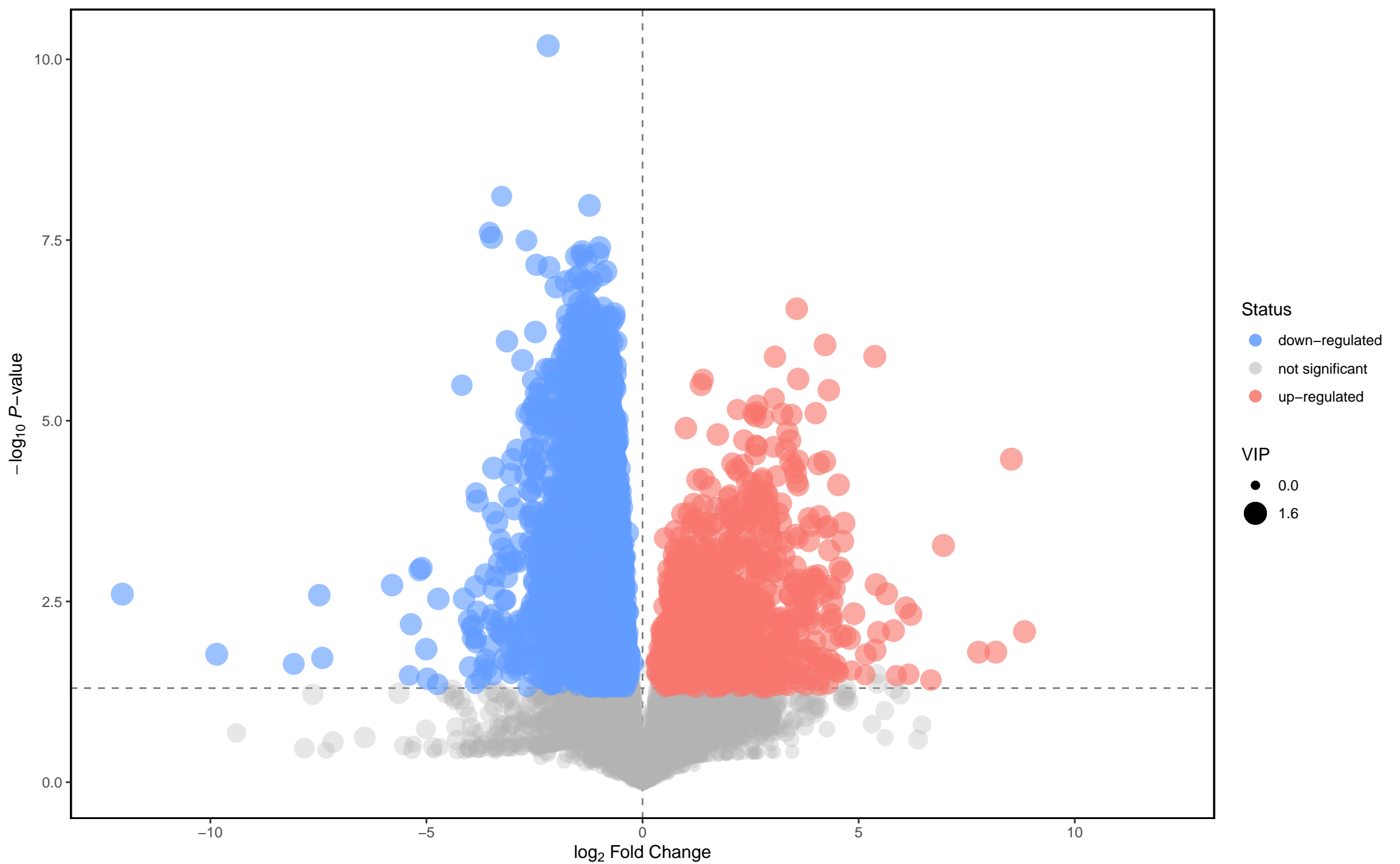

Supplement: Supplementary file 4 [file Data_Sheet_2.ZIP › raw data for cells/raw data/POS-for metabolomics/Statistical Analysis/HX VS NX/volcano plot.pdf]

Figure 1 A=NX, B= HX


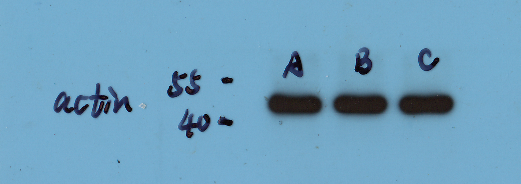


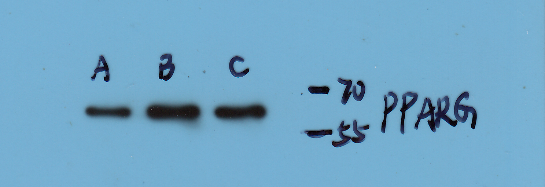


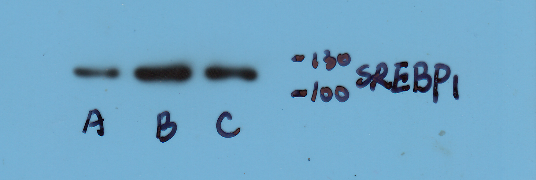

Supplement: Supplementary file 4 [file Data_Sheet_2.ZIP › raw data for cells/raw data/WB.docx]
