# Supplementary material for: Multi-Omics Analysis of Mammary Metabolic Changes in Dairy Cows Exposed to Hypoxia
Source: Front Vet Sci. 2021 Oct 14;8:764135. doi: 10.3389/fvets.2021.764135 (PMC8553012; doi:10.3389/fvets.2021.764135)
Supplement: Supplementary file 4 [file Data_Sheet_2.ZIP › raw data for cells/raw data/BODIPY staining.docx]

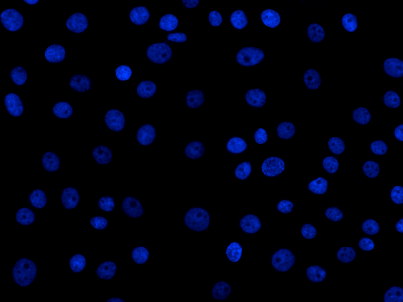

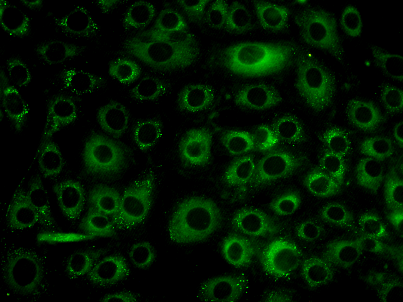

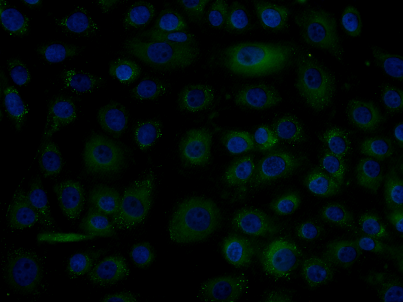


NX DAPI BODIPY MERGE


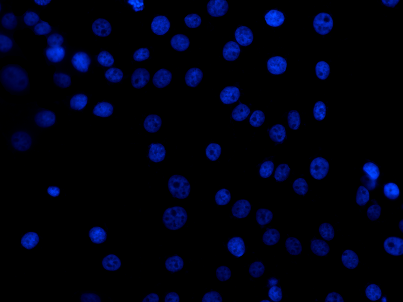

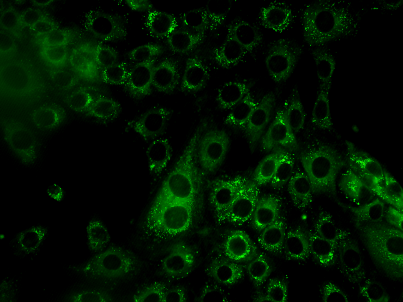

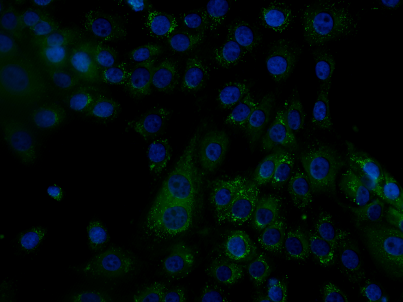


HX DAPI BODIPY MERGE


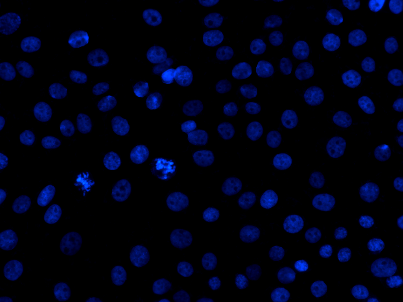

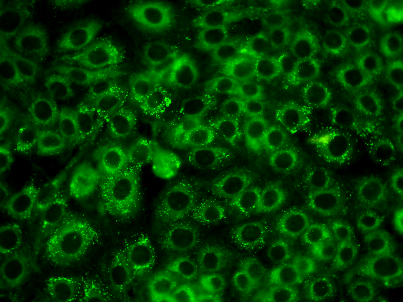

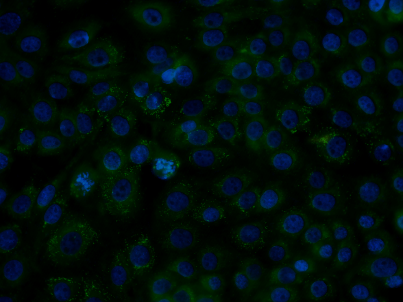


NX DAPI BODIPY MERGE


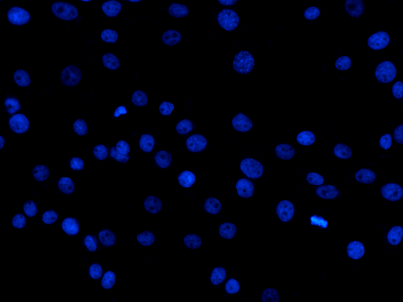

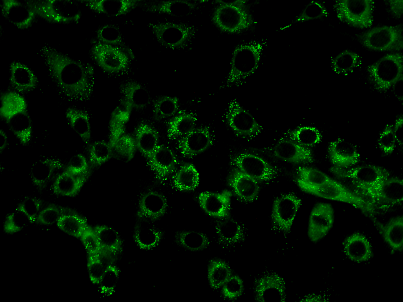

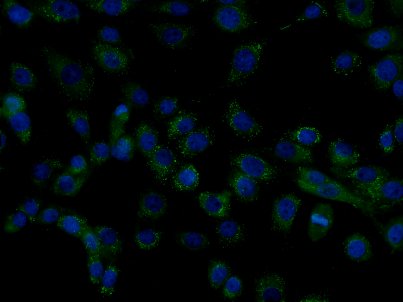


HX DAPI BODIPY MERGE


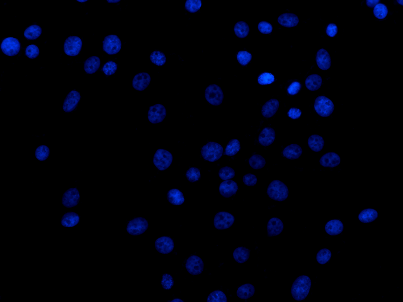

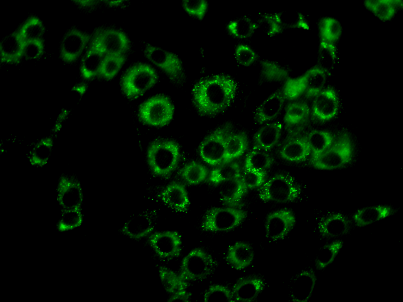

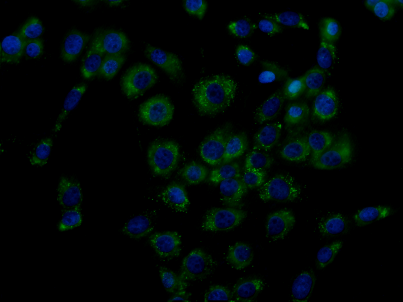


HX+shAGPAT2 (DAPI) BODIPY MERGE
